# CD206 modulates the role of M2 macrophages in the origin of metastatic tumors

**DOI:** 10.7150/jca.91944

**Published:** 2024-01-21

**Authors:** Hui Li, Ying-Qi Miao, Li-Ping Suo, Xi Wang, Yi-Qing Mao, Xue-Hui Zhang, Na Zhou, Jun-Rui Tian, Xiu-Yan Yu, Tong-Xia Wang, Yan Gao, Hong-Yan Guo, Zheng Zhang, Dian-Sheng Ma, Hong-Xia Wu, Yan-Wei Cui, Xi-Liang Zhang, Xiao-Chun Chi, Yu-Chi Li, David M. Irwin, Gang Niu, Huan-Ran Tan

**Affiliations:** 1 Department of Pharmacology, School of Basic Medical Sciences, Peking University Health Science Center, Beijing, China.; 2 Department of Obstetrics and Gynecology, Peking University Third Hospital, Beijing, China.; 3 Peking University First Hospital, Beijing, China.; 4 Donggang Central Hospital, Donggang, China.; 5 Department of Human Anatomy, Histology and Embryology, School of Basic Medical Sciences, Peking University Health Science Center, Beijing, China.; 6 Kellbenx Inc., Great River, NY, USA.; 7 Department of Laboratory Medicine and Pathobiology, University of Toronto, Toronto, Canada.; 8 Beijing N&N Genetech Company, Beijing, China.

**Keywords:** CD206, M2 macrophages, metastatic tumors, apoptosis, polarization

## Abstract

Tumor metastasis is a key factor affecting the life of patients with malignant tumors. For the past hundred years, scientists have focused on how to kill cancer cells and inhibit their metastasis *in vivo*, but few breakthroughs have been made. Here we hypothesized a novel mode for cancer metastasis. We show that the phagocytosis of apoptotic tumor cells by macrophages leads to their polarization into the M2 phenotype, and that the expression of stem cell related as well as drug resistance related genes was induced. Therefore, it appears that M2 macrophages have "defected" and have been transformed into the initial "metastatic cancer cells", and thus are the source, at least in part, of the distal tissue tumor metastasis. This assumption is supported by the presence of fused cells with characteristics of both macrophage and tumor cell observed in the peripheral blood and ascites of patients with ovarian cancer. By eliminating the expression of CD206 in M2 macrophages using siRNA, we show that the growth and metastasis of tumors was suppressed using both *in vitro* cell line and with experimental *in vivo* mouse models. In summary, we show that M2 macrophages in the blood circulation underwent a "change of loyalty" to become "cancer cells" that transformed into distal tissue metastasis, which could be suppressed by the knockdown of CD206 expression.

## Introduction

Most patients with malignant tumors lose their lives due to the effects of distant metastasis of these tumors 1, however, the mechanisms generating these distal metastases remain unresolved. Based on our studies, we hypothesize that distant cancer metastases may not originate from tumor cells that travel through the blood circulation, but instead are due to M2 macrophages that have “changes in loyalty” and become "cancer cells" as a result of their interactions with primary cancer cells.

Antitumor immunotherapy activates the immune system to fight tumors and strengthen immune responses 2. Macrophages are a major component of the immune response to tumor cells and differentiate into subtypes M1 and M2 3. M2 macrophages, which are also known as tumor associated macrophages (TAMs), have characteristics similar to tumor precursors, promote tumor growth and metastasis, and are the most diverse immune cell population in the tumor microenvironment 4. Many available antitumor immunotherapies 5 cause malignant tumors to undergo apoptosis, leading to apoptotic tumor cells, or their active bodies (including exosomes), being phagocytized by macrophages 6. In this study we found that after phagocytosis, macrophages become polarized into the M2 type where a "defection" may occur. These M2 macrophages then may become, at least in part, the "distal tumor metastatic cells" due to this "defection", and thus, TAM2 are important factors in the distal metastasis of tumors.

M2 macrophages play a central role in tumor progression and resistance to cancer treatment 7. CD206 is a highly specific marker for M2 macrophages 8 that lacks intracellular signaling motifs, thus is generally considered to function as an endocytic receptor for the internalization of extracellular substances for clearance or antigen presentation 9. Recent work has shown that CD206 is active in the immune response by directly regulating the activity of other types of immune cells 10, although conflicting results on the relationship between the regulation of CD206 expression and tumor metastasis has been reported 11, 12. A promising therapeutic strategy involving TAMs is to induce a reprogramming from immunosuppressive M2-like TAMs to M1-like macrophages 13. Here we explore the role of M2 macrophages in tumor metastasis due to their “change of loyalty”. We also examined the role of M2 macrophages in assisting tumor metastasis from the perspective of macrophage function, along with the consequence of the loss of CD206 in tumor development and metastasis. Our previous analyses of CTCs (circulating tumor cells) from patients with tumor metastasis showed that both primary tumor cells and fusion cells possessing tumor cell and M2 macrophage markers can be detected in the blood of patients 14. Fusion cells might be metastatic cancer cells with a special affinity to specific tissues. Our previous studies found that the metastatic cancer cells might be M2 macrophage cells that acquired tumor genes 14, which we called tumor-like M2 macrophages (TM2) or tumacrophage. Normally, with tumors, a large number of monocytes gather at a tumor site. We found that monocytes can be polarized by tumor cells into M2 macrophages that non-selectively phagocytize apoptotic tumor cells leading to the creation of fusion cells (which we named tumacrophages) that can be detected in the peripheral blood. Here, we found that the cell cycle and proliferative ability of M2 macrophages was not affected by siRNA knock-down of CD206 expression, but that the abundance of IL-10, CCL17, CCL22, MMP2 and MMP9 secreted into the supernatant by these M2 macrophages decreased significantly. Interestingly, inhibiting the expression of CD206 in M2 macrophages resulted in a significant reduction in the weights of tumors and the number of metastases in tumor-bearing mice. These observations support the hypothesis that the distant metastasis of a tumor is due to the migration of macrophages that have "defected" and have become tumor cells. Therefore, M2 macrophages are an important cradle for metastasis and are a potential target for tumor treatment.

## Materials and Methods

### Animals, patients and ethical approvals

Adult female Balb/c, male ICR and male athymic nude (Balb/c nu-nu) mice, 6-8 weeks of age, were purchased from the Animal Center of the Peking University Health Science Center (Beijing, China). Animals were housed in a pathogen-free animal facility. Standard laboratory chow and water were provided *ad libitum*. Peripheral blood and ascites were drawn from healthy human donors and ovarian cancer patients treated at Peking University Third Hospital (Beijing, China). Informed consent was obtained from all human subjects. All research was performed in accordance with the declaration of Helsinki. The Biomedical Ethics Committee of Peking University approved all animal (Permit #LA2016069) and human (Permit # IRB00001052-16022) experiments. All experiments were carried out in accordance with the ARRIVE guidelines.

### Differentiation of THP1 monocytes into macrophages

THP1 monocytes (ATCC® TIB-202™) were cultured in RPMI 1640 (Life Technologies, USA) media supplemented with 10% (v/v) heat inactivated fetal bovine serum (FBS) (Biological Industries, USA), 100 U/ml penicillin, and 100 μg/ml streptomycin (Amresco® Life Science, USA) and maintained at 37°C in a humidified 5% CO_2_ atmosphere. THP1 monocytes were seeded at a density of 5×10^5^ cells/ml in 6-well or 24-well plates (Corning, New York, NY, USA) and stimulated for polarization into T-M0 macrophages by the addition of 200 nM PMA. After 24 h, the original medium was discarded and replaced with RPMI-1640 medium supplemented with 10% FBS containing a final concentration of 100 ng/mL LPS and IFN-γ and cultured for 24 h to obtain T-M1 macrophages or with RPMI-1640 medium supplemented with 10% FBS containing a final concentration of 20 ng/mL IL-4 and IL-13 and cultured for 24 h to obtain T-M2 macrophages.

### Harvesting and polarization of mouse bone marrow-derived macrophages

Bone marrow-derived macrophages were obtained from 6~8-week-old Balb/c mice. Mice were sacrificed by cervical dislocation. Clean bones were obtained by dissection and soaked in PBS. Bone marrow was aspirated from the samples using a 5 mL syringe with bone marrow cells collected into a 15 mL centrifuge tube. Cells were filtered through a 70 µm (200 mesh) cell strainer and collected by centrifugation at 1000 rpm for 10 min. The cell pellet was resuspended and cultured with DMEM complete medium containing a final concentration of 50 ng/mL M-MCSF (PeproTech, Cranbury, USA). Conditioned medium was changed on day 3 after plating, and mature macrophages were harvested on day 7. Bone marrow-derived macrophages were polarized with different cytokines. To obtain M-M1 macrophages, the original medium was discarded, and RPMI-1640 medium supplemented with 10% FBS containing a final concentration of 100 ng/mL LPS and IFN-γ was added to the culture and incubated for 24 h. For M-M2 macrophages, RPMI-1640 medium supplemented with 10% FBS containing a final concentration of 20 ng/mL IL-4 was added and cultured for 24 h.

### Harvesting and polarization of human macrophages

Fresh human patient venous blood (5 ml) was collected in EDTA anticoagulation tubes. Mononuclear cells were isolated from the blood by centrifugation through a Ficoll Paque-plus (GE Healthcare, USA) density gradient. Isolated cells were pelleted and resuspended in RPMI-1640 media and cultured for 3 days, to obtain human monocytes (H-M). Polarized macrophages (H-M2) were generated by treating these cells with 20 ng/ml IL-4 and culturing for 3 additional days.

### Knockdown of CD206 expression using small interfering RNA (siRNA)

Small interfering RNA (siRNA) sequences targeting CD206, and a negative control RNA (NC), were synthesized by Guangzhou Rui Bo Biotechnology Co., Ltd. (Guangzhou, China). siRNA and NC mimics were transfected into T-M2 and M-M2 macrophages using the riboFECT CP Transfection Kit (RiboBio, Guangzhou, China) according to the manufacturer's protocol. Efficiency of interference by the siRNA and NC was assessed using RT‒qPCR and Western blotting 48 h after transfection. The target sequences for the siRNA were 5'-GTGTGACCATGTATTCAAA-3' (HS1), 5'-CAACCAGGATGCCGAATCA-3' (HS2), 5'-GGATCGCCCTGAACAGTAA-3' (HS3), 5'-GTGGTATGCAGACTGCACC-3' (MS1), 5'-GCAAGCATTTGTTACCTAT-3' (MS2), and 5'-GCATGAAGCAGAGACATAT-3' (MS3).

### RT-qPCR analysis

Total RNA was extracted using TRIzol (Invitrogen, New York, NY, USA) and transcribed into cDNA with a RevertAid First Strand cDNA Synthesis Kit (K1622, Thermo Scientific, MA, USA). RT‒qPCR was performed using the Applied Biosystems StepOne™ Real-Time RT‒PCR System with PowerUp™ SYBR® Green Master Mix (Applied Biosystems, Foster city, USA). Primers are listed in Supplementary Table 1. β-Actin was used as the internal control to correct for variation in cDNA content among samples. No nonspecific amplification product was observed for any of the reactions, as determined from an analysis of the dissociation curves. Data were normalized to the β-actin expression levels. Relative mRNA abundance of the target genes was calculated by the 2-ΔΔCt method, where Ct represents the threshold cycle.

### Cell immunofluorescence

THP1 or mouse bone marrow-derived macrophages (BMDMs) were seeded onto glass confocal dishes (Cellvis, Hangzhou, China) and stimulated to polarize into macrophages. Polarized cells were washed with cold phosphate-buffered saline (PBS), and then fixed in 4% paraformaldehyde at room temperature for 15 min. Cells were blocked with 5% bovine serum albumin (BSA) (Amresco® Life Science, USA) in PBS for 1 h at room temperature. Diluted primary antibodies against CD68, CD163, CD204 or CD206 (Abcam, USA) were added and incubated overnight at 4°C. Primary antibodies are listed in supplementary Table 2. Primary antibodies were detected using diluted secondary antibody that was incubated with the cells for 1 h at room temperature. After incubation, excess secondary antibody was removed, and 200 μl of DAPI (Solarbio® Life Sciences, Beijing, China) was added and incubated for 10 min at room temperature in the dark. Cells were then washed 3 times with PBS and observed under a confocal microscope (Leica TCS SP8 MP FLIM).

### Western blot analysis

Cells were lysed with 100 μL RIPA lysis buffer (Applygen, Beijing, China) on ice for 60 min and then centrifuged at 12,000 rpm for 20 min at 4°C to pellet protein. The protein pellet was resuspended in 20μL PBS. Protein concentration was measured by the BCA (Applygen, China) protein determination method. The same amount of total protein (40 μg) was loaded onto 6% or 10% polyacrylamide gels for sodium dodecyl sulfate‒polyacrylamide (SDS-PAGE) gel electrophoresis. Proteins were transferred to polyvinylidene difluoride membranes (PVDF). PVDF membranes were blocked with 5% BSA for 2 h and then incubated with diluted primary antibodies overnight at 4°C. Primary antibodies are listed in supplementary Table 2. After washing three times with TBST, the PVDF membranes were incubated with the secondary antibody (A21010, Abbkine, Wuhan, China) at room temperature for 1 hour. Proteins were detected by an ECL system (Bio-Rad, USA) and analyzed using ImageJ (1.5.3) to determine abundance. Each experiment was repeated three times.

### ELISA analysis

Cell supernatants were collected and centrifuged at 1000 rpm for 10 min. ELISAs were then performed on the supernatant according to the kit's instructions. ELISA kits are listed in supplementary Table 3.

### Cell proliferation assay

Cell proliferation was measured using the CCK-8 or MTS assay (Yeasen, China). Cells were inoculated at a density of 5000 cells per well in 96-well plates. After the cells adhered to the plate surface, the original medium was replaced with different types of conditioned media, and 6 auxiliary wells were set for each group. At 0, 12, 24, 36 and 48 hours of cell culture, 10 μL of CCK-8 or MTS solution was added to a well according to the manufacturer's instructions and incubated at 37°C for 2 h. The OD value was measured at 450 nm using a microplate reader (Bio-Rad, USA). Each experiment was repeated three times.

### Tumor transplantation into mice

Mouse breast cancer tumor models were constructed by inoculating cells into the armpit of the right forelimb or tail vein of mice. The mouse breast cancer cell line 4T1-luc (2 × 10^5^), suspended in 200 μl PBS, was injected subcutaneously into the armpit of the right forelimb or injected into the tail vein of Balb/c mice (female, 6-8 weeks old). The mouse breast cancer tumor model constructed by inoculating cells into the tail vein and was imaged on the 8th day after inoculation and on day 16 for subcutaneous inoculation. Mice were killed by dislocation of their cervical vertebra. Tumor size was calculated from the shorter diameter (W) and longer diameter (L) of the tumor, and tumor volume calculated according to the formula v=w^2^ × L/2. Breast tumor, lung, and liver tissue were removed and weighed, embedded in paraffin, sectioned, stained with HE and histochemistry examination was conducted under a microscope.

### CTC and circulating fusion cells

CTC and circulating fusion cells were examined using peripheral blood from patients and healthy volunteers. The study group consisted of 82 EOC patients, 27 benign ovarian tumor patients and 16 healthy female donors. All patients and healthy donors gave written informed consent before participation. Blood samples (5 ml) were placed in Vacuette EDTA tubes (BD Biosciences, USA) and stored at 4°C. Magnetic powder (Dynabeads M-450 Tosylactivated, Invitrogen, USA)-labelled monoclonal antibodies (Abcam, United Kingdom) were added to 5 ml of blood and incubated at 4°C for 30 minutes. Monoclonal antibodies for EpCAM, HER2 and MUC1 were used to isolate circulating tumor cells; antibodies against CD68 and CD163 were used to isolate circulating macrophage/fusion cells. Cells were lysed using 200 µl of lysis buffer, and mRNA was isolated according to the Dynabeads mRNA DIRECT Kit instructions and then reverse transcribed into cDNA using the Sensitive Reverse Transcription Kit (Qiagen, Germany). The abundance of ovarian cancer-related genes (EpCAM, HER2, MUC1, WT1, P16 and PAX8) was quantified by multiplex RT‒qPCR, and PCR products were visualized using an Agilent 2100 Bioanalyzer (Agilent Technologies, USA) with DNA 1000 LabChips (Agilent Technologies, USA).

### Observation of phagocytosis of macrophages using Cell Tracker

Normal MCF-7 cells and mouse primary cultured hepatocytes were washed twice with PBS, digested with 0.25% trypsin, and centrifuged at 1000 rpm for 5 min to collect cells. Cell tracker probe dye was diluted with DMEM complete medium to obtain working solutions. Macrophages were treated with green CMFDA and MCF-7 or mouse primary cultured hepatocytes were treated with red CMTMR for 30 min under 5% CO_2_ at 37°C. Red CMTMR labelled MCF-7 cells or mouse primary cultured hepatocytes were mixed with green CMFDA macrophage at a 2:1ratio (MCF-7/ hepatocytes: macrophage). After co-culture for 3 h, cells were fixed with 3.7% paraformaldehyde at room temperature for 15 min and photographed under an Olympus inverted microscope.

### Flow cytometry

Cells to be tested were washed twice with PBS, digested with 0.25% trypsin, collected in a centrifuge tube, and pelleted by centrifugation at 1000 rpm for 5 min. Conditioned medium was discarded. Cell pellets were resuspended in 1 ml PBS, and cell concentrations adjusted to 5 × 10^5^ cells/tube. Appropriate marker (Hoechst 33342 for cell cycle analysis, PI and Annexin V for apoptosis analysis, or antibody for surface marker analysis) was added to each tube and incubated in the dark for 0.5 h at 4℃. Unbound antibody was washed off and the cells were analyzed on a BD FACS Calibur within 1 h of staining.

### Transwell assay

Cell migration and invasion capacity were assessed using a Transwell assay. The bottom chambers of the transwell apparatus were filled with 600 µL of different conditioned media. In the top chamber of the Transwell apparatus, 5×10^4^ cells in 100 uL of media without FBS was added. Cells that migrated to, or invaded, the other side of the chamber were subjected to 4% paraformaldehyde fixation for 30 min and detected using 0.1% crystal violet staining. Images were acquired using an inverted microscope and migrating or invading cells were quantified using ImageJ (1.5.3). This experiment was repeated three times.

### Wound healing assay

Cells were inoculated at a density of 2×10^5^ cells per well into 6-well plates with an indicated conditioned medium and allowed to adhere to the surface. When the cells reached 80% confluence, a straight trace was scratched in the middle of each well with a 10-μL pipette tip. Cells were then rinsed three times with PBS, and new conditioned medium was added. Cells were cultured for 24 h and imaged with an inverted microscope at 0 h and 24 h. Using Image J software (1.5.3), the scratch area of each group was measured, and the ratio of the scratch area at 24 h and 0 h was calculated.

### Extraction of exosomes from MCF-7-cell conditioned medium

Exosomes were isolated from cell culture media from logarithmically growing MCF-7 cells by centrifugation at 100,000 g at 4°C for 70 min. The pellet was resuspended in PBS and named MCF7 exo. MCF7 exo was stored at -80°C until use. A drop of exosome liquid resuspended in PBS was added to a 200 meshes carbon-coated copper net for 1 min. After staining with 2% phosphotungstic acid (pH 7.0) for 1 min, the exosomes were observed and photographed with a JEM-1400 plus transmission electron microscope at 120 kV.

### Statistical analysis

Experimental data generated in these experiments are expressed as mean ± standard deviation (SD). The significance of the difference comparisons was assessed by one-way analysis of variance (one-way ANOVA). SPSS 22.0 software was used for the data analysis, and P<0.05 was considered to be statistically significant.

## Results

### Downregulation of CD206 in mouse bone marrow-derived M2 macrophages (M-M2) inhibits growth and metastasis of breast cancer cells in mice

It has previously been shown that high expression of CD206 is correlated with a poor prognosis in breast 11, colorectal 15 and liver cancer 16. However, the role of CD206 in M2 cells in the promotion of breast cancer remains unclear. To examine the functions of CD206 in M2 cells, we established models of T-M2 (human M2 macrophages derived from THP1 cells) and M-M2 (mouse M2 macrophages derived from mouse bone marrow macrophage cells) cells that have characteristics of M2 cells as reflected by their biomarker CD206 (Supplementary Fig. 1). First, we designed three mouse-specific siRNAs (MS1, MS2 and MS3) to knock-down CD206 expression in mouse M2 macrophage cells and selected the MS3 siRNA that showed the greatest downregulation of CD206 in M-M2 cell (Supplementary Fig. 2) for the following experiments.

**Figure 1:**
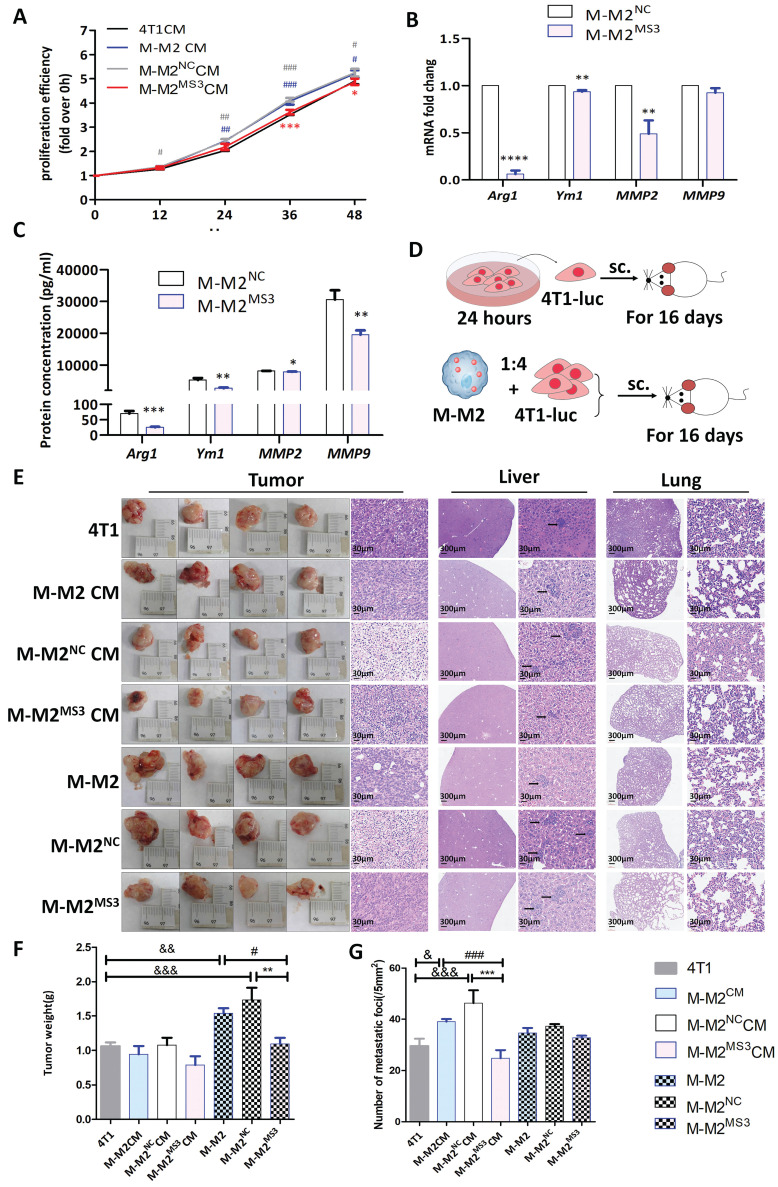
Downregulation of CD206 in mouse bone marrow-derived M2 macrophages (M-M2) inhibits the growth of breast cancer cells in mice. (**A**) The CCK8 assay was used to evaluate the proliferative ability of MCF-7 cells exposed to conditioned medium (CM) from MCF-7, M-M2, M-M2NC and M-M2MS3 (experimental group with interfered expression of CD206) cells. Fold change in proliferation was calculated compared to 0 h (* indicates statistical significance vs M-M2NC CM, *: P<0.05, ***: P<0.001; # indicates statistical significance vs 4T1 CM, #: P<0.05, ##: P<0.005, ###: P<0.001) (n =3). (**B**) Abundance of Arg1, Ym1, MMP2 and MMP9 mRNA in M-M2 cells transfected with MS3 or NC siRNA for 48 h were quantified by RT-qPCR (* indicates statistical significance vs NC, **: P<0.005, ****: P<0.0001). (n = 3). (**C**) ELISA was used to measure the abundance of Arg1, Ym1, MMP2 and MMP9 protein in M-M2 supernatant (* indicates statistical significance vs NC, *: P<0.05, **: P<0.005) (n =3). (**D**) Experimental scheme for the cancer model with a subcutaneous inoculation of tumor cells into Balb/cBalb/c nu-nu mice. M-M2^NC^ CM and M-M2^MS3^ CM groups: 4T1-Luc cells were treated with conditioned medium from M-M2^NC^ and M-M2^MS3^ cells for 24 hours and then inoculated subcutaneously into Balb/cBalb/c nu-nu mice. M-M2^NC^ and M-M2^MS3^ groups: M-M2^NC^ and M-M2^MS3^ cells were mixed with 4T1-Luc cells at a ratio of 1:4 and inoculated subcutaneously into Balb/cBalb/c nu-nu mice. (**E**) Representative images of subcutaneous tumors and HE staining of tumor sections. HE staining of mouse liver and lung tissue sections. Black arrows indicate metastases to the liver or lung. Mice were sacrificed 16 days after subcutaneous inoculation with tumor cell according to the experimental procedure shown in Fig. 1D. (n =4). (**F**) Tumor weights from mice sacrificed 16 days after subcutaneous inoculation with tumor cells according to the experimental procedure shown in Fig 1D. Subcutaneous tumors were harvested and weighed (&&: P<0.005, &&&: P<0.001; #: P<0.05, **: P<0.005). (**G**) Counts of the numbers of metastases obtained from multiple randomly selected HE stained liver tissue section visual fields (&: P<0.05, &&&: P<0.001; ###: P<0.001; ***: P<0.001) (n =4).

We assessed CCK-8 levels to study the effect of M-M2 macrophages with decreased CD206 abundance on the proliferation of 4T1 breast cancer cells. Significantly decreased levels of CCK-8, and thus proliferative activity, was found in 4T1 cells treated with M-M2MS3 CM (conditioned medium collected from M-M2 cells with knocked down CD206 expression by the MS3 siRNA) at 36 (P<0.005 vs M-M2 CM, P<0.001 vs M-M2NC CM) and 48 (P<0.05) hours compared with cells treated with M-M2 CM or M-M2NC CM (Fig. 1A). In contrast, the proliferative activity of 4T1 cells in M-M2 CM (P<0.005) and M-M2NC CM (P<0.001) were significantly increased in comparison to cells in 4T1CM. These results suggest that M-M2 macrophages promote the proliferation of breast cancer 4T1 cells and that this capacity was decreased after downregulating CD206 expression (Fig. 1A).

Arg1 17 and Ym1 18 are fundamental features of M2 macrophages, with the secretion of Arg1 19, Ym1 20, MMP2 21 and MMP9 22 by macrophages promoting the metastasis of tumor cells. To study the secretion of Arg1, Ym1, MMP2 and MMP9 by M-M2 macrophages after downregulating CD206 expression, RT-qPCR and ELISA were used to assess their mRNA and protein levels, respectively.

RT-qPCR results showed that, compared to the NC (negative control) group, the mRNA abundance for Arg1 (P<0.0001), Ym1 (P<0.005) and matrix metalloproteinase MMP2 (P<0.005) were significantly downregulated in M-M2 cells treated with MS3 whereas no significant change for MMP9 was detected (Fig. 1B). Analysis of protein abundance in the M-M2 cell culture supernatants by ELISA showed that the abundance of Arg1 (P<0.001), Ym1 (P<0.005), MMP2 (P<0.05) and MMP9 (P<0.005) were significantly reduced in cells treated with MS3 compared to NC (Fig. 1C). These results indicate that the downregulation of CD206 inhibits the expression and secretion of Arg1, Ym1, MMP2 and MMP9 in M-M2 macrophages.

To examine whether the proliferative activity of tumor cells parallel the downregulation of CD206 in M2 macrophages, an *in vivo* 4T1-Luc cancer model was used. 4T1-Luc cells were first cultured with conditioned medium from M2 macrophages with (M-M2^MS3^ CM) or without (M-M2^NC^ CM) CD206 knockdown or co-cultured with M2 macrophages with (M-M2^MS3^) or without (M-M2^NC^) CD206 knockdown for 24 hours, and then inoculated into the armpit of the left forelimb of Balb/c mice. Mice were sacrificed by dislocation of their cervical vertebra 16 days after the injections and the resulting tumors were examined (Fig. 1D). Compared to tumors in mice that received control 4T1 cells, tumor weights in the M-M2 (P<0.005) or M-M2^NC^ (P<0.001) mice were significantly increased (Fig. 1E, F). Tumor weights in M-M2^MS3^ mice were significantly decreased compared with those in M-M2 or M-M2^NC^ mice (P<0.05 vs M-M2 CM, P<0.005 vs M-M2^NC^ CM) (Fig. 1E, F). Pathological examination of HE stained liver tissue showed that the number of metastases in the M-M2 CM (P<0.005) and M-M2^NC^ CM (P<0.001) mice were significantly increased compared to those receiving 4T1 cells. In contrast, the number of metastatic foci in the liver of M-M2^MS3^ CM mice (P<0.001 vs M-M2 CM, P<0.001 vs M-M2^NC^ CM) was significantly reduced compared with M-M2 CM or M-M2^NC^ CM mice. No metastases were found in the examined lung tissue samples (Fig. 1E, G).

**Figure 2:**
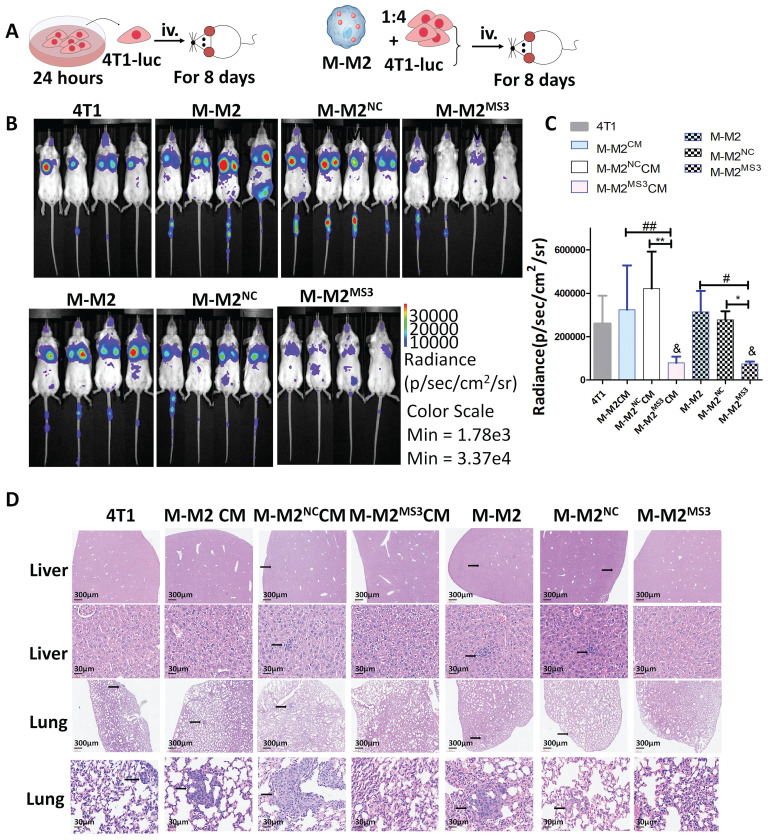
Downregulation of CD206 in mouse bone marrow-derived M2 macrophages (M-M2) inhibits metastasis of breast cancer cells in mice. (**A**) Experimental scheme for the cancer model with tumor cells injected into the tail vein of Balb/cBalb/c nu-nu mice. M-M2 CM, M-M2^NC^ CM and M-M2^MS3^ CM groups: 4T1-Luc cells were treated with conditioned medium from M-M2, M-M2^NC^ or M-M2^MS3^ cells, respectively, for 24 hours and then injected into the tail vein of Balb/cBalb/c nu-nu mice. M-M2, M-M2^NC^ and M-M2^MS3^ groups: M-M2, M-M2^NC^ and M-M2^MS3^ cells were mixed with 4T1-Luc cells at a ratio of 1:4 and injected into the tail vein of Balb/cBalb/c nu-nu mice. (**B-C**) Representative images of small animal imaging (**B**) and quantification of lung fluorescence density (**C**) 8 days after tail vein injection with tumor cells (& indicates statistical significance vs 4T1, &: P<0.05, *: P<0.05, **: P<0.005; #: P<0.05, ##: P<0.005) (n=4). (**D**) Metastases were detected in mice that were sacrificed 8 days after tail vein injection with tumor cells. Liver and lung tissue sections of the mice were stained with HE. Black arrows indicate metastases to the liver or lung (* indicates statistical significance, P < 0.05) (n=4).

A second mouse breast cancer model was also used to study the effect of M2 macrophages on breast cancer tumor metastasis after CD206 knockdown. For this model, 4T1-Luc cells treated with M-M2^NC^ or M-M2^MS3^ conditioned medium (CM) or were co-cultured with M-M2^NC^ or M-M2^MS3^ cells were inoculated into Balb/c mice through the caudal vein (Fig. 2A). Whole body fluorescence intensity (representing metastatic tumor cells) of the M-M2^MS3^ CM mice was significantly lower than in those receiving M-M2 CM or M-M2^NC^ CM cells (P<0.005 vs M-M2 CM, P<0.005 vs M-M2^NC^ CM) after 8 days, and M-M2^MS3^ mice had significantly lower levels of fluorescence than those receiving M-M2 or M-M2^NC^ cells (P<0.05 vs M-M2, P<0.05 vs M-M2^NC^) (Fig. 2B,C). No metastases were seen by light microscope examination of HE stained liver and lung tissue sections from the mice that received M-M2^MS3^ CM or M-M2^MS3^ cells, while an increased number of metastases were observed in both the liver and lungs of mice that were inoculated with all other types of cells (Fig. 2D).

These results demonstrate that conditioned medium from M-M2 macrophages with CD206 knockdown inhibit the growth and metastasis of 4T1 breast cancer cells in mice.

### Downregulation of CD206 affects the expression of cytokines and the activity of the TLR4/Myd88/NF-κB signaling in human M2 macrophages leading to the suppression of proliferation, migration and invasion of MCF-7 cells

To further examine the function of CD206 in M2 macrophages, three human-specific siRNAs (HS1, HS2 and HS3) were designed to interfere with the expression of CD206 in human T-M2 macrophages. Low abundance of CD206 in T-M2 macrophages was observed 48 h after HS1 treatment (Supplementary Fig. 3).

**Figure 3:**
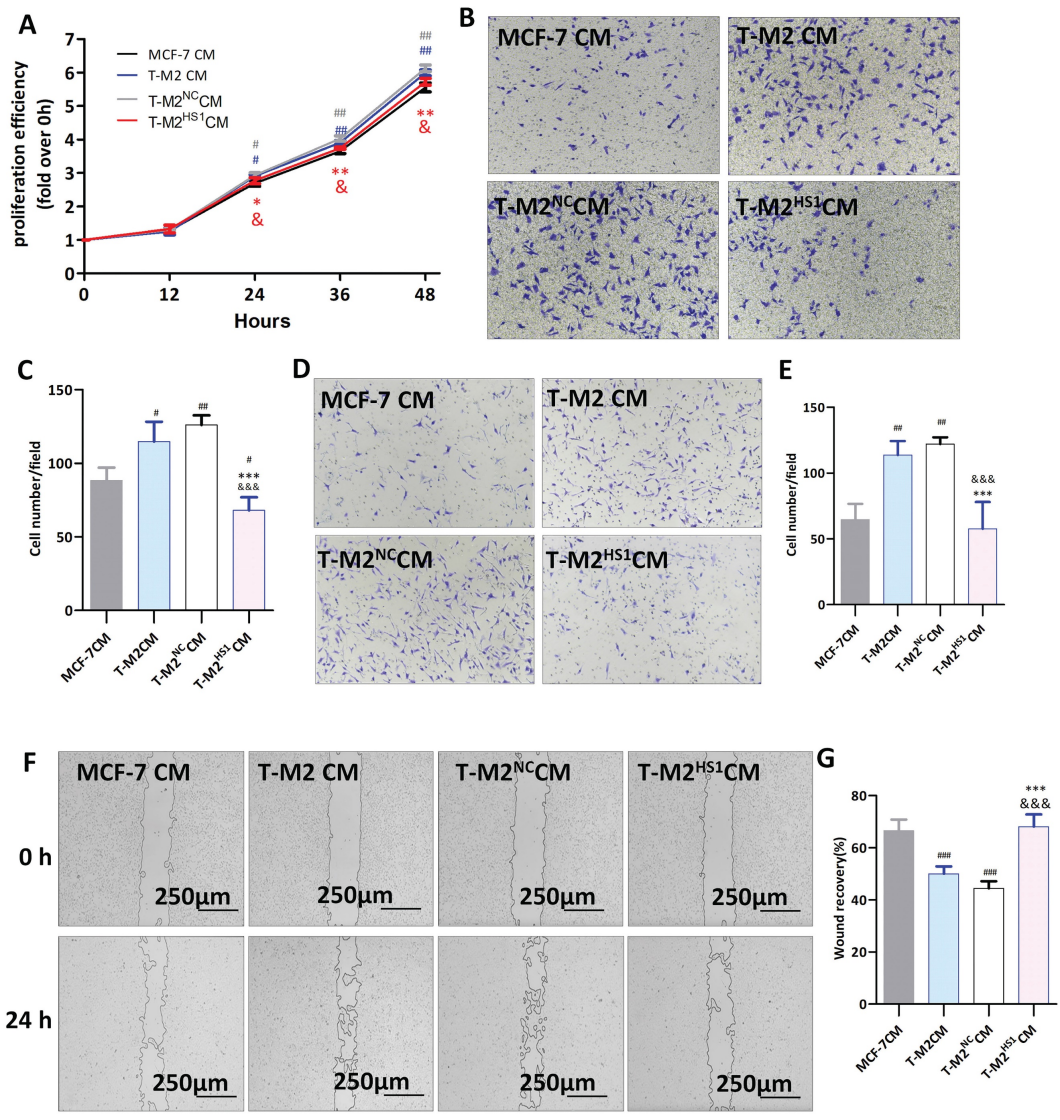
Downregulation of CD206 inhibits the proliferation, migration and invasion of human MCF-7 cells. (**A**) The CCK8 assay was used to evaluate the proliferation of MCF-7 cells cultured with conditioned medium from MCF-7 (MCF-7 CM), T-M2 (T-M2 CM) and T-M2 cells transfected with negative control (M-M2^NC^ CM) and anti CD206 (T-M2^HS1^ CM) siRNA. Proliferation efficiency is compared to 0 h (* indicates statistical significance vs T-M2^NC^ CM, *: P<0.05, **: P<0.005; # indicates statistical significance vs MCF-7 CM, #: P<0.05, ##: P<0.005; & indicates statistical significance vs T-M2 CM, P<0.05) (n = 3). (**B-C**) Migration of MCF-7 cells was measured using a Transwell chamber. Conditioned medium from MCF-7 (MCF-7 CM), T-M2 (T-M2 CM), T-M2^NC^ (M-M2^NC^ CM) and T-M2^HS1^ (T-M2^HS1^ CM) cells were placed in the lower Transwell chamber. MCF-7 cells were cultured in the upper chamber. The number of migrating MCF-7 cells were counted at 24 h, with representative images (**B**) and cell counts (**C**) shown (*** indicates statistical significance vs T-M2^NC^ CM, P<0.001; # indicates statistical significance vs MCF-7 CM, #: P<0.05, ##: P<0.005; &&& indicates statistical significance vs T-M2 CM, P<0.001) (n = 3). (**D-E**) Conditioned medium of MCF-7 (MCF-7 CM), T-M2 (T-M2 CM), T-M2^NC^ (M-M2^NC^ CM) and T-M2^HS1^ (T-M2^HS1^ CM) cells were placed in the Transwell lower chamber. MCF-7 cells were cultured with Matrigel in the upper chamber for 24 h, with representative images (**D**) and cell counts (**E**) of cells that migrated from the upper chamber to the bottom layer shown (*** indicates statistical significance vs T-M2^NC^ CM, P<0.001; ### indicates statistical significance vs MCF-7 CM, P<0.005; &&& indicates statistical significance vs T-M2 CM, P<0.001) (n =3). (**F-G**) Representative images of the scratch wound healing assay for MCF-7 cells cultured with conditioned medium from MCF-7 (MCF-7 CM), T-M2 (T-M2 CM), T-M2^NC^ (M-M2^NC^ CM) and T-M2^HS1^ (T-M2^HS1^ CM) cells for 24 h. Photos were taken under a microscope at 0 h and 24 h (**F**), and the percentage of scratch area covered by MCF-7 cells (**G**) was calculated using ImageJ software (*** indicates statistical significance vs T-M2^NC^ CM, P<0.001; ### indicates statistical significance vs MCF-7 CM, P<0.001; &&& indicates statistical significance vs T-M2 CM, P<0.001) (n =3).

To study the role of CD206 in T-M2 macrophages in the proliferation, migration and invasion of MCF-7 breast cancer cells, MCF-7 cells were treated with conditioned media (CM) from T-M2 cells transfected with siRNA against CD206 (T-M2^HS1^ CM), control siRNA (T-M2^NC^ CM), no siRNA (T-M2 CM), or CM from MCF-7 cells (MCF-7 CM). Proliferation of MCF-7 cells was measured using the CCK-8 assay at 12, 24, 36 and 48 hours. Our results showed that the proliferation rate of MCF-7 cells treated with T-M2 CM and T-M2^NC^ CM was significantly higher than in cells treated with MCF-7 CM, and the proliferation of cells cultured with T-M2^HS1^ CM was significantly lower than in cells cultured in T-M2 CM or T-M2^NC^ CM (Fig. 3A). In the cell migration assay, MCF-7 cells treated with both T-M2 CM (P<0.05) and T-M2^NC^ CM (P<0.005) showed significantly increased levels of metastasis, an effect that was reversed in cell treated with conditioned media from T-M2 cells transfected with the HS1 siRNA (T-M2^HS1^ CM) (Fig. 3B, C). The invasive activity was further examined in Transwell chambers coated with Matrigel. As seen with the migration study, MCF-7 cells treated with both T-M2 CM (P<0.005) and T-M2^NC^ CM (P<0.005) showed significantly increased levels of invasion, while the invasive potential of cells cultured in T-M2^HS1^ CM were not different from those cultured in MCF-7 CM (Fig. 3D, E).

We also evaluated the metastatic potential of MCF-7 cells using the scratch healing experiment. Similar to the results above, MCF-7 cells treated with both T-M2 CM (P<0.001) and T-M2^NC^ CM (P<0.001) showed significantly increased levels of wound healing, while T-M2^HS1^ CM had no effect (Fig. 3F, G). These results indicate a potential for T-M2 macrophages to promote the proliferation and metastasis of MCF-7 breast cancer cells, which is eliminated when CD206 expression is downregulated.

**Figure 4:**
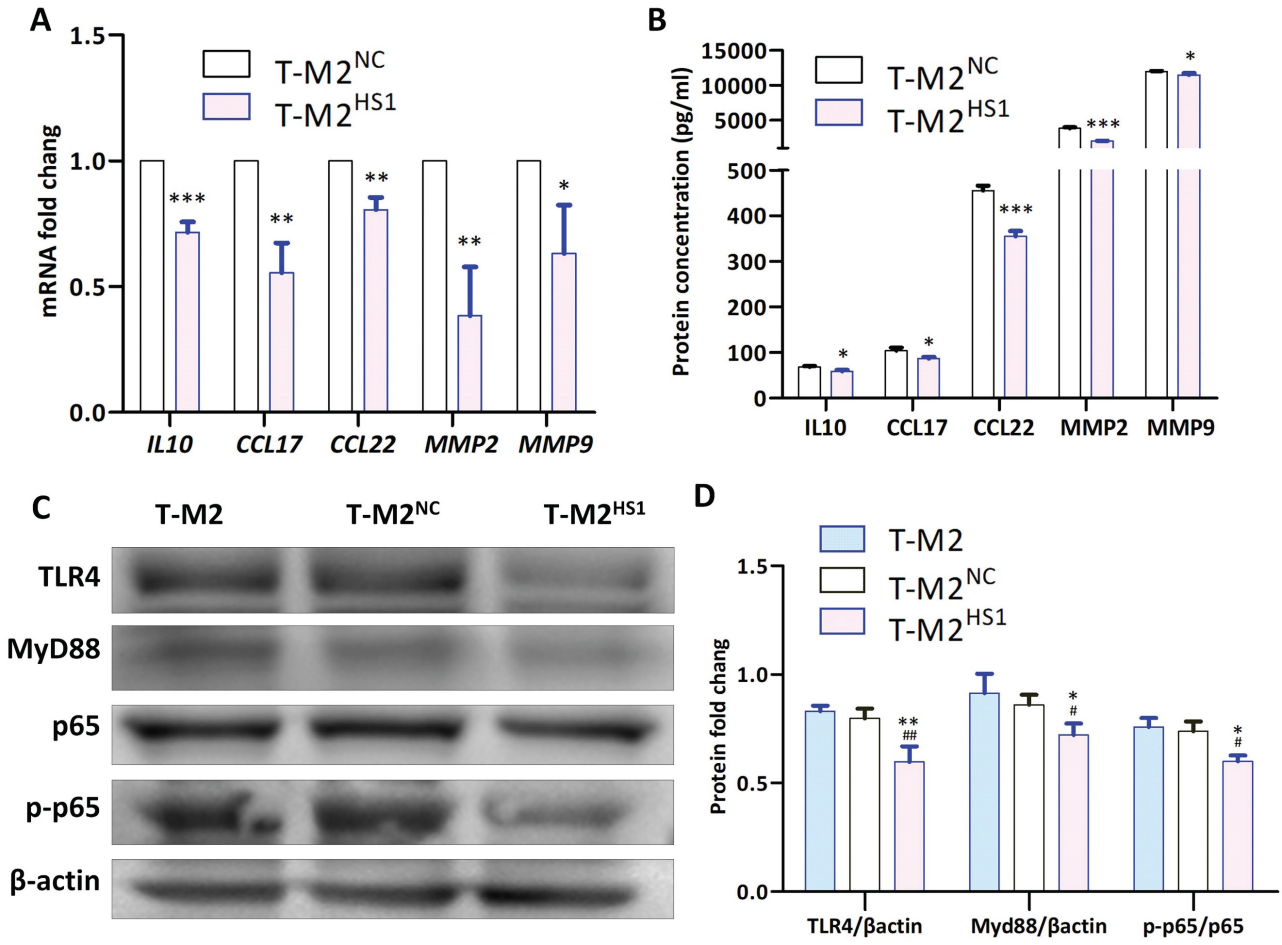
Downregulation of CD206 downregulate the human TLR4/MyD88/NF-κB signaling pathway. (**A**) Abundance of IL10, CCL17, CCL22, MMP2 and MMP9 mRNA in T-M2 and HS1-transfected T-M2 cells quantified by RT-qPCR (* indicates statistical significance vs NC, *: P<0.05, **: P<0.005, ***: P<0.001) (n =3). (**B**) Supernatant from T-M2 cells was collected to measure the abundance of secreted IL10, CCL17, CCL22, MMP2 and MMP9 by ELISA. T-M2 cells were transfected with HS1 or NC siRNA for 48 h (* indicates statistical significance vs NC, *: P<0.05, ***: P<0.001) (n =3). (**C-D**) Representative Western blots (**C**) and their quantification (**D**) for TLR4, MyD88 and p-P65/P65 abundance in T-M2 cells transfected with HS1 or NC siRNA for 48 h. TLR4 and MyD88 were normalized against actin, p-P65 were normalized against total P65 (* indicates statistical significance vs T-M2^NC^, P<0.05; # indicates statistical significance vs T-M2, *: P<0.05, **: P<0.005; # indicates statistical significance vs T-M2, #: P<0.05, ##: P<0.005) (n =3).

To study the mechanism by which CD206 regulates the functions of T-M2 macrophages, RT-qPCR, ELISA and Western blotting were used to measure the expression of M2 macrophage-related factors/genes after knockdown of CD206 expression. Results of an RT-qPCR analysis showed that the mRNA abundance of IL-10 (P<0.001), CCL17 (P<0.005), CCL22 (P<0.005), MMP2 (P<0.005) and MMP9 (P<0.05) in T-M2^HS1^ cells vs. T-M2^NC^ cells was significantly reduced (Fig. 4A), which were paralleled by significant decreases in protein levels detected by ELISA in the cell supernatants (IL-10 (P<0.05), CCL17 (P<0.05), CCL22 (P<0.001), MMP2 (P<0.001) and MMP9 (P<0.05)) (Fig. 4B). In addition, Western blot analysis showed that, compared with the cells transfected with the NC siRNA, TLR4 (P<0.005), MyD88 (P<0.05) and phosphorylated p-p65 NF-κB (P<0.05) protein levels in T-M2^HS1^ cells were significantly decreased (Fig. 4C, D).

These results show that the downregulation of CD206 expression weakens the tumor-promoting effect of T-M2, probably via inhibition of the TLR4/p65 signaling pathway.

### Expression of tumor-related genes in TAMs circulating in blood is closely associated with tumor progression

**Figure 5:**
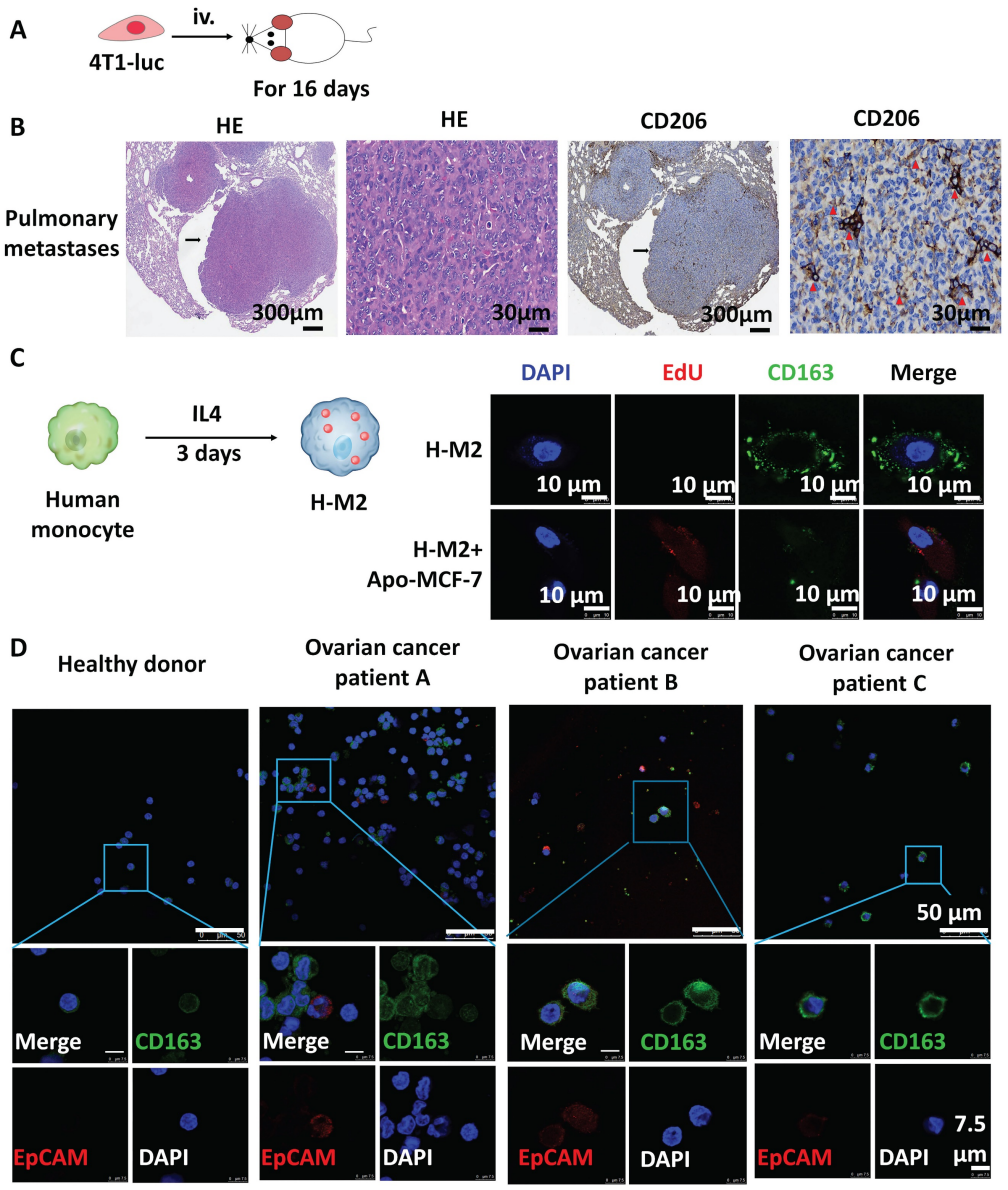
Expression of tumor-related genes in tumor-associated macrophages. (**A**) Experimental scheme for the cancer model. (**B**) Representative images of HE stained and immunohistochemistry for CD206 in mouse lung tissue sections 16 days after tail vein injection with 4T1-luc. Black arrows indicate metastases to the lung. Red triangles indicate immunohistochemically positive staining areas. (**C**) Scheme showing the experimental strategy used to induce polarization of human peripheral blood mononuclear cells into M2 macrophages (H-M2). Apoptotic MCF-7 (apo-MCF-7) cells were obtained by treatment with 0.3 mM hydrogen peroxide for 12 hours and were labeled with EdU, and then cocultured with H-M2 cells (immunofluorescence labeled with CD163) for an additional 3 days. Cells were photographed by confocal microscopy. (**D**) Representative images of immunofluorescence staining for CD163 (green) and EpCAM (red) in nucleated cells isolated through Ficoll gradients from peripheral blood of healthy donor and three ovarian cancer patients.

Tumor cells and tumor DNA are present in the circulating blood of patients during tumor progression 23. A previous study from our group revealed that in addition to tumor cells, some specific macrophages were also present in the circulating blood of the tumor patients 14. We found in this study that the CD206 positive cells could be detected by immunohistochemical staining in metastatic 4T1 tumors 16 days after the cells had been injected into mice (Fig. 5A, B). To further study the activity of human TAMs in human tumor patients and their ability to phagocytize apoptotic tumor cells, monocytes isolated from the peripheral blood of healthy volunteers were treated with IL4 for 3 days to induce differentiation into M2 macrophages. The resulting M2 macrophages displayed high expression of CD163, a marker for M2 macrophages. We found that these polarized M2 macrophages could phagocytize apoptotic MCF-7 tumor cells when they were cocultured with apoptotic MCF-7 cells (Fig. 5C). This data suggests that M2 macrophages may also be able to phagocytize apoptotic tumor cells *in vivo*, thereby potentially transforming M2 macrophages into tumor cells in the blood of tumor patients. To further examine this hypothesis, the interactions between M2 macrophages and tumor cells in patients was studied. The phenotypes of nucleated cells in the peripheral blood obtained from three patients with ovarian cancer and from healthy donors (as control) were analyzed. Interestingly, phagocytic/fusion cells with the epithelial marker EpCAM and the macrophage marker CD163 were only observed in blood cells from patients with ovarian cancer (Fig. 5D).

**Figure 6:**
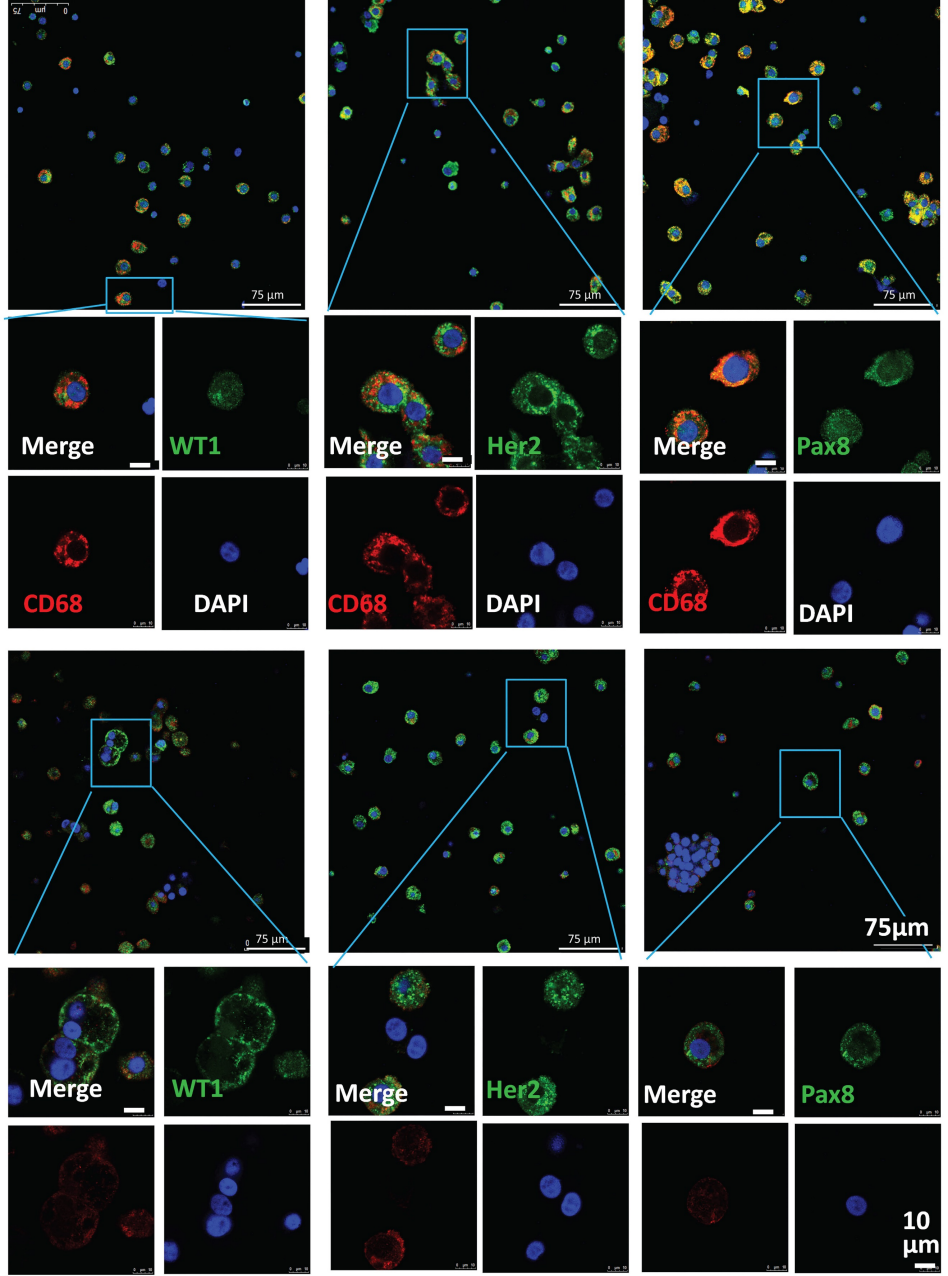
Phenotypic characterization of ascities cells from cancer patients. Representative images of immunofluorescence staining for CD68 (red), CD11b (red), WT1 (green), Her2 (green) and Pax8 (green) in ascites cells from ovarian cancer patients obtained by centrifugation.

Ascites cells with tumor-related markers (WT1, HER2 or Pax8) and macrophage markers (CD68 or CD11b) were also observed in confocal images from these patients (Fig. 6), suggesting that the phagocytic/fusion cells with epithelial and macrophage characteristics were also present in the ascites of patients with ovarian cancer. These observations confirm that fusion cells with characteristics of both macrophages and tumor cells were present in the blood and ascites of ovarian cancer patients. These phagocytic/fusion cells might have been formed by macrophages that phagocytized apoptotic tumor cells or tumor cell exosomes and may be involved in tumor metastasis.

**Figure 7:**
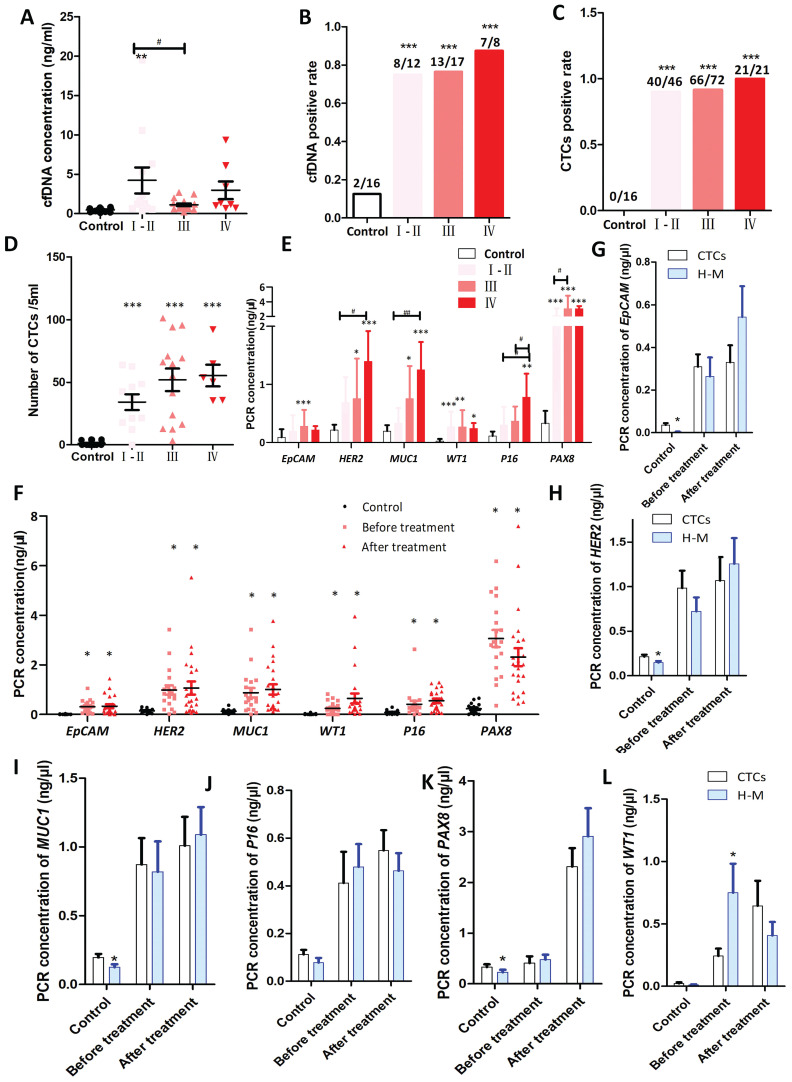
Tumor-associated macrophages in circulating blood are closely associated with tumor development. (**A-B**) Amount of circulating free DNA in peripheral blood (**A**) and the positive rate for the presence of cfDNA (**B**) in cancer patients of different stages of disease. Samples from healthy donors were used as control. I, II, III and IV represent stages of ovarian cancer (* indicates statistical significance vs control, **:P<0.005, ***:P<0.001; # indicates statistical significance, P<0.05). (**C-E**) Numbers of circulating tumor cells (CTCs) in the peripheral blood of ovarian cancer patients of different stages. CTCs were isolated with immunomagnetic beads against tumor-associated proteins (EpCAM, HER2 and MUC1) and quantified by multiplex PCR. The positive rate of for CTCs in cancer patients (**C**), number of CTCs (**D**), and the abundance of genes used for CTC identification (**E**). Samples from healthy donors were used as control. I, II, III and IV represent the stage of ovarian cancer (* indicates statistical significance vs control, *: P<0.05, **: P<0.005, ***: P<0.001; # indicates statistical significance, #: P<0.05, ##: P<0.005). (**F**) Tumor macrophage fusion cells (H-M) in the peripheral blood circulation of ovarian cancer patients isolated by immunomagnetic beads against macrophage-related proteins (CD68 and CD163) and quantified by multiplex PCR. Abundance of ovarian cancer-related genes in tumor macrophage fusion cells (H-M) (* indicates statistical significance vs control, P < 0.05). (**G-L**) Difference in the abundance of ovarian cancer-related genes in macrophage fusion cells and CTCs in the peripheral blood circulation of patients with ovarian cancer (* indicates statistical significance vs CTCs, P<0.05).

Circulating blood tumor cells (CTCs) and free DNA (cfDNA) may carry tumor information 24. To explore the relationship between CTCs, cfDNA, and phagocytic/fusion cells with M2 markers, and their effect on tumor progression, we first compared the total number and positivity rate for extracellular cfDNA from patients with newly diagnosed ovarian cancer at different stages and compared them with 16 healthy donors. Results showed that the total amount of cfDNA (P<0.005 at stage I/ II) (Fig. 7A) and the positivity rate for cfDNA (P<0.001) (Fig. 7B) are significantly higher in ovarian cancer patients at each stage than in healthy volunteers. No significant difference in the positivity rate was seen between cancer patients in the different stages (Fig. 7B).

We then compared the numbers and positivity rates for CTCs in patients with newly diagnosed ovarian cancer at different stages. The results show that in newly diagnosed ovarian cancer, the positivity rate for CTCs in healthy volunteers was 0.00% for stage I, 86.95% for stage II, 91.66% for stage III, and 100% for stage IV (Fig. 7C). The number of circulating tumor cells in each cancer stage were higher than seen in healthy volunteers (P<0.001) (Fig. 7D). The abundance of PCR products for the tumor-related markers in each stage of ovarian cancer was higher than in healthy volunteers (Fig. 7E).

To explore the abundance of transcripts for tumor-related genes in phagocytic/fusion cells with epithelial and macrophage characteristics, CD163 and CD68 antibodies were used to isolate macrophages from human peripheral blood. RT-qPCR shows that mRNA levels for the tumor-related markers EpCAM, HER2, MUC1, WT1, p16 and Pax8 were significantly higher in macrophages from the peripheral blood of ovarian cancer patients, both before and after treatment, than that from healthy volunteers (P<0.05), although there was no significant difference between levels seen in patients before and after treatment (Fig. 7F). The abundance of mRNAs for tumor-related genes in CTCs and phagocytic/fusion cells was also examined. No significant difference in the abundance of EpCAM, HER2, MUC1, P16 and PAX8 mRNAs were seen between CTCs and macrophages in ovarian cancer patients before treatment (Fig. 7G-K). The exception was that the abundance of WT1 in macrophages was significantly higher than in CTCs (P<0.05) (Fig. 7L). No significant difference in the abundance of HER2, MUC1, WT1, P16 and PAX8 mRNAs was seen in CTCs and macrophages before and after treatment, although the abundance of EpCAM in macrophages was significantly higher after treatment than in CTCs (P<0.05) (Fig. 7G-L).

### Macrophages selectively phagocytize apoptotic tumor cells, in preference to live cells, and phagocytosis stimulates macrophage proliferation and tumor promotion

**Figure 8:**
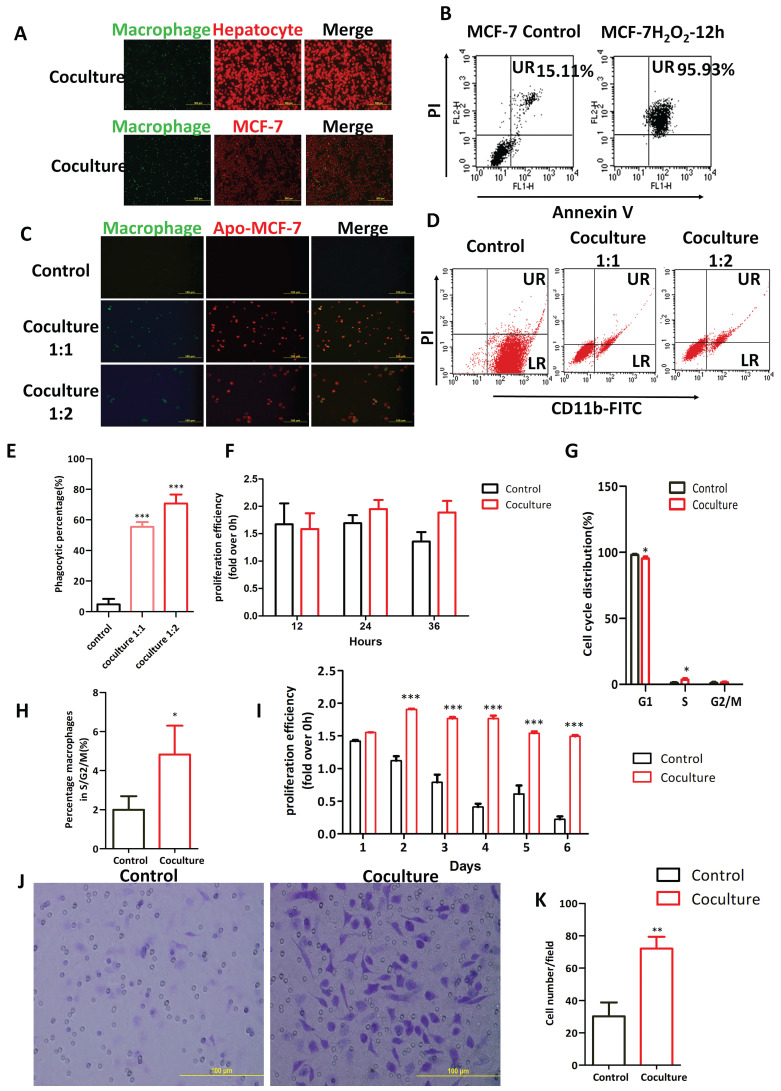
Mouse macrophages selectively phagocytize apoptotic tumor cells rather than live cells, and phagocytosis stimulates mouse macrophages proliferation and tumor promotion. (**A**) Representative fluorescent images of mouse peritoneal macrophages cocultured with mouse hepatocytes and human MCF-7 cells. Cells were cocultured for 3 hours after labeling macrophages with the green Cell Tracker probe and target cells with red Cell Tracker probe. (**B**) Apoptosis of MCF-7, when treated with 0.3 mM hydrogen peroxide for 12 hours, was measured by flow cytometry and Annexin V/PI staining (n = 3). (**C-E**) Representative fluorescent images (**C**) and flow images (**D**) of mouse peritoneal macrophages cocultured with PI-labeled apo-MCF-7 at ratios of 1:1 and 1:2 for 3 h. Apoptotic MCF-7 (apo-MCF-7) were obtained by treating cells with 0.3 mM hydrogen peroxide for 12 hours. Macrophages were incubated with FITC-conjugated anti-CD11b antibody. UR (PI^+^, CD11b^+^): macrophages that have engulfed apo-MCF-7cells. LR (PI^-^, CD11b^+^): macrophages. Phagocytosis ratio was calculated as UR/(UR + LR)×100% (**E**) (n=3). (**F-I**) Cell cycle distributions (**F-G**) were measured using flow cytometry. Cells were seeded into 96-well plates, and cell proliferation (**H-I**) was assayed using the MTS method. Coculture group: mouse peritoneal macrophages cocultured with apoptotic MCF-7 cells (apo-MCF-7) for 3 hours. Control group: macrophages cultured alone (n =3). (**J-K**) Macrophages cocultured with or without apoptotic MCF-7 were inoculated into the upper Transwell chamber in DMEM without FBS. The lower chamber was macrophage conditioned medium containing 20% serum. After culturing for 24 hours, crystal violet staining (**J**) was performed to count the number of cells (**K**) that migrated through the Transwell chamber membrane (* indicates statistical significance vs control, *: P<0.05, **: P<0.005, ***: P<0.001) (n =3).

Macrophages are important immune cells *in vivo*. Their main function is to phagocytize foreign substances and secrete cytokines to trigger subsequent immune responses. Macrophages in the tumor microenvironment have multiple interactions with tumor cells. To observe phagocytosis, mouse macrophages and nonapoptotic target cells were labeled with cell tracker probes (green fluorescence labelled mouse peritoneal macrophages and red fluorescence labelled target cells) and cocultured for 3 h. No colocalization of red and green fluorescence was observed under a fluorescence microscope (Fig. 8A) indicating that mouse peritoneal macrophages did not phagocytize homologous primary cultured hepatocytes or cells of the human breast cancer cell line MCF-7.

The phagocytic activity of mouse macrophages towards apoptotic tumor cells was examined in an environment that mimicked a local macrophage infiltration of the microenvironment of breast cancer. Over 95% of MCF-7 cells become apoptotic after 12 hours of exposure to H_2_O_2_ (Fig. 8B). To distinguish apoptotic MCF-7 cells and macrophages, we labeled these cells with PI (red) and a FITC-conjugated anti-CD11b antibody (green), respectively. Macrophages that engulfed apoptotic MCF-7 cells should then be double stained (CD11b^+^/PI^+^, green/red). Immunofluorescent examination of these cells revealed colocalization of red and green fluorescence, with red apoptotic MCF-7 cells wrapped around membrane-bound macrophages labelled green by CD11b antibodies (Fig. 8C, Merge). The proportion of the CD11b^+^ cells that were also PI^+^ was approximately 55% when measured by flow cytometry when the coculture (macrophage: apoptotic MCF-7) ratio was 1:1 (P<0.001), which increased to approximately 70% when the coculture ratio was 1:2 (P<0.001) (Fig. 8D, E).

Proliferation, cycle distribution and migration of the mouse peritoneal macrophages after phagocytosis of apoptotic tumor cells were then analyzed. Proliferation rates, as determined by the MTS assay, of mouse peritoneal macrophages after 24h and 36 h coculture with apoptotic MCF-7 cells was not significantly higher than in cells that were not cocultured (Fig. 8F). The lifespan of primary cultured macrophages, as measured by the MTS assay, was extended by 6 days when they were exposed to apoptotic MCF-7 cells. Cell numbers, without apoptotic MCF-7 cells treatment, began to decline after 1 day, and only approximately 10% of cells survived at day 6.

However, when macrophages were cocultured with apoptotic MCF-7 cells, within 2 days they displayed rapid proliferation (Fig. 8I) that was then followed by a decline yielding no significant decrease in the numbers of viable macrophages at day 6. To determine the reasons for the rapid proliferation of mouse peritoneal macrophages upon phagocytosis of apoptotic tumor cells, cell cycle progression of these cells was analyzed by a flow cytometer analysis. Data from this experiment showed that, compared with control cells, the proportion of cells in G0/G1 phase was significantly decreased (P<0.05) and the proportion of cells in S/G2/M phases increased significantly (P<0.05) in the coculture group (Fig. 8G, H). To explore the migratory potential of macrophages cocultured with or without apoptotic MCF-7, cells from the cocultures (+/- apoptotic MCF-7 cells) were resuspended in serum-free culture medium in the upper chamber of a Transwell apparatus. The lower chamber contained 20% serum. After 24 hours the numbers of macrophages that migrated to the lower chamber were counted. Macrophages that were cocultured with apoptotic MCF-7 cells displayed a significantly higher numbers of migrating cells compared to control cells (P<0.005) (Fig. 8J, K).

**Figure 9:**
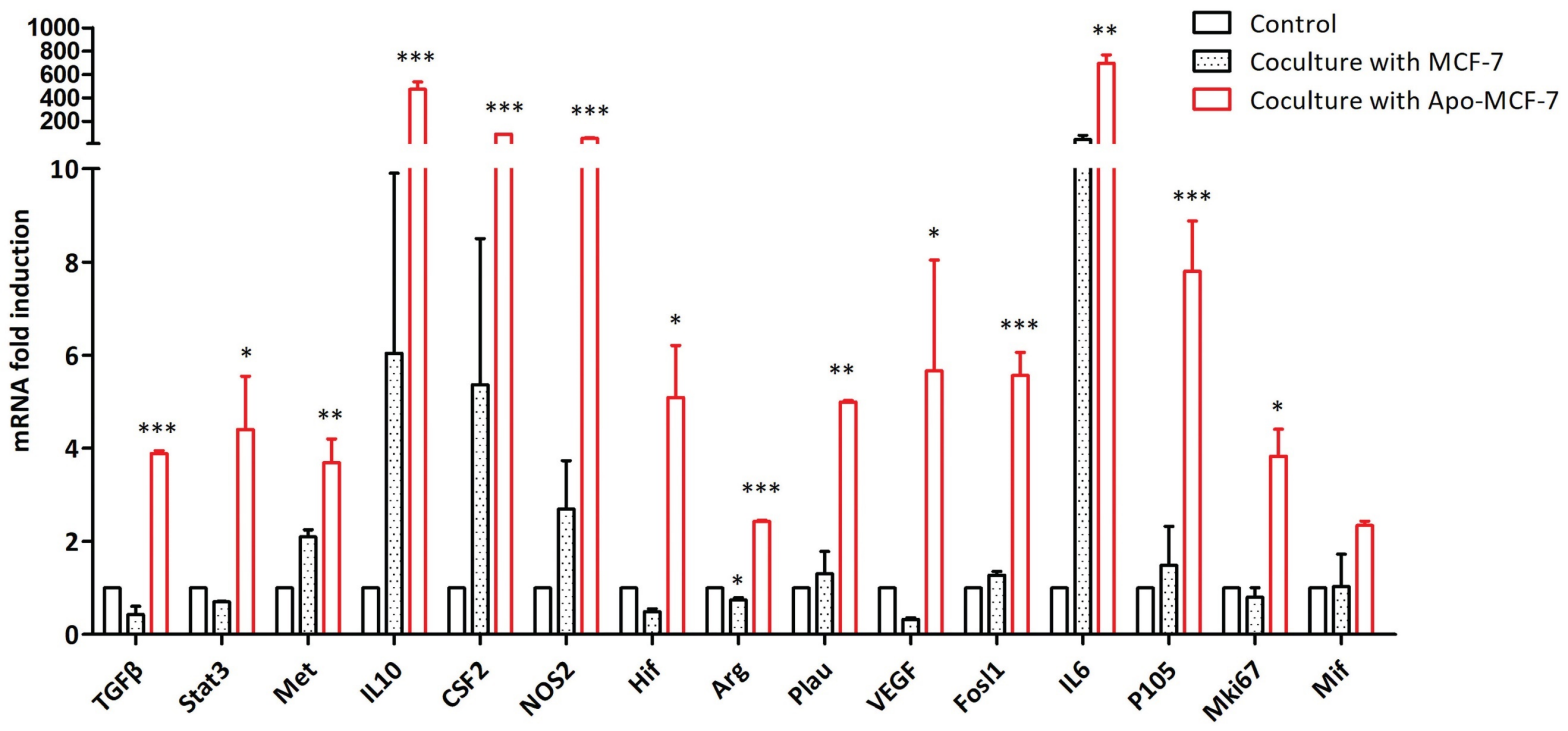
Changes in gene expression in mouse macrophages after phagocytosis of apoptotic tumor cells. Expression of selected genes in macrophages co-cultured with apoptotic or non-apoptotic MCF-7 cells, or with no MCF-7 cells (control) (* indicates statistical significance vs control, *: P<0.05, **: P<0.005, ***: P<0.001).

We used RT-qPCR to study the changes in the gene expression profiles of mouse macrophages that have phagocytosed apoptotic MCF-7 cells. Higher expression of Stat3, cell proliferation-related factors (Met, P105, Fosl1 and Mki67), tumor-promoting-related factors (Arg, HIF, NOS2, TGFβ, Plau and VEGF) and cytokines (IL10, IL6 and CSF2) was found in macrophages that phagocytized apoptotic MCF-7 cells compared with cells that were not occulted with MCF-7 cells. On the other hand, macrophages cocultured with nonapoptotic MCF-7 cells showed no significant changes in the abundance of the mRNAs for the other genes compared to the control cells (Fig. 9).

### Phagocytosis of MCF-7 exosomes induces polarization human macrophages to the M2 type

As an important intercellular signal transmission pathway, exosomes allow communication between tumor cells and macrophages. MCF-7 exosomes were characterized by ultracentrifugation, transmission electron microscopy and particle size analysis. Laser confocal microscopy of T-M0 macrophages revealed that DiD-labelled exosomes were evenly distributed in the cytoplasm of these cells (Supplementary Fig. 4).

**Figure 10:**
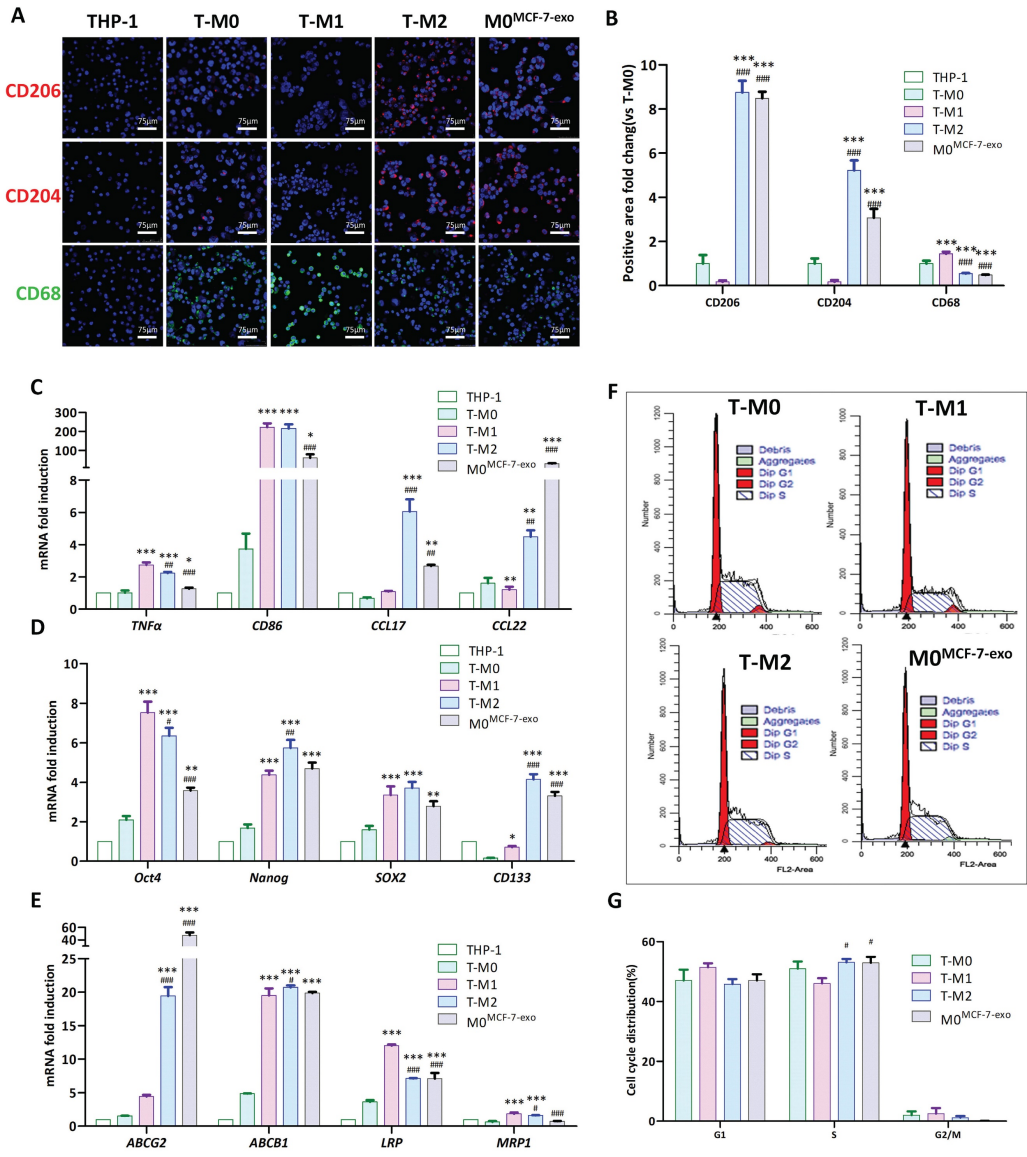
Phagocytosis of MCF-7 exosomes induces polarization of human macrophages to the M2 type. (**A-B**) Representative immunofluorescent images (**A**) and fold changes in fluorescence positive areas (**B**). M0^MCF-7-exo^ cells: T-M0 macrophages cocultured with MCF-7 exosomes (*** indicates statistical significance vs T-M0, P<0.001; ### indicates statistical significance vs T-M1, P<0.001) (n =3). (**C-E**) RT-qPCR was used to quantify the mRNA abundance for selected genes in T-M0, T-M1, T-M2 and M0^MCF-7-exo^ cells (T-M0 cells cocultured with MCF-7 exosomes) (* indicates statistical significance vs T-M0, *: P<0.05, **: P<0.005, ***: P<0.001; # indicates statistical significance vs T-M1, #: P<0.05, ##: P<0.005, ###: P<0.001) (n =3). (**F-G**) Representative flow cytometry images (**F**) and cell cycle distributions (**G**) of T-M0, T-M1, T-M2 and M0^MCF-7-exo^ cells (T-M0 cells cocultured with MCF-7 exosomes) (* indicates statistical significance vs T-M0, P<0.05; # indicates statistical significance vs T-M1, P<0.05) (n =3).

To explore the impact of MCF-7 exosomes on the promotion of polarization of M0 macrophages into the M2 phenotype, surface proteins of T-M0 (human M0 macrophages derived from THP1 cells) macrophages treated with MCF-7 exosomes (named M0^MCF-7-exos^) were examined. Immunofluorescence showed that the abundance of the characteristic T-M1 macrophage protein CD68 in M0^MCF-7-exo^ cells was reduced compared to T-M0 cells, while the abundance of the characteristic M2 macrophage proteins CD206 (P<0.001) and CD204 (P<0.001) were significantly increased (Fig. 10A, B). RT-qPCR showed that TNF-α (P<0.001) and CD86 (P<0.001) mRNAs were in lower abundance in M0^MCF-7-exo^ cells than in T-M1 macrophages, while CCL17 (P<0.005) and CCL22 (P<0.001) were at higher abundance (Fig. 10C). These results suggest that the exposure of M0 macrophages to MCF-7 exosomes induces a polarization into M2 macrophages.

To examine the effect of MCF-7 exosomes on self-renewal and the replication potential of T-M0 macrophages, the expression of stem cell-related genes Oct4, Nanog, SOX-2 and CD133 were examined. RT-qPCR data showed that mRNAs for Nanog, Sox-2, Oct4 and CD133 were significantly more abundant in M0^MCF-7-exo^ cells compared to T-M0 macrophages, suggesting that exposure to MCF-7 exosomes enhanced self-renewal and replication of M0 macrophages (Fig. 10D).

M2 macrophages may also contribute to tumor recurrence and drug resistance. To examine this, the expression of drug resistance-related genes ABCG2, ABCB1, LRP and MRP1 were examined. The results of an RT-qPCR analysis showed that the mRNA levels for the drug resistance-related genes ABCG2 (P<0.001), ABCB1 (P<0.001) and LRP (P<0.001) were significantly more abundant in M0^MCF-7-exos^ cells than in T-M0 macrophages, suggesting that exposure to MCF-7 exosomes enhanced drug resistance in M0 macrophages (Fig. 10E).

Flow cytometry was also used to analyze the cell cycle distribution of T-M0, T-M1, T-M2 and M0^MCF-7-exos^ cells (Fig. 10F). Flow cytometry showed that the proportion of T-M1, T-M2, and M0^MCF-7-exo^ cells in the G0/G1, S, and G2/M phases of the cell cycle did not significantly change compared with T-M0 macrophages, suggesting that MCF-7 exosomes did not affect the cell cycle of M0 macrophages (Fig. 10G).

### Phagocytosis of MCF-7 exosomes by human macrophages promotes the migration and proliferation of tumor cells

**Figure 11:**
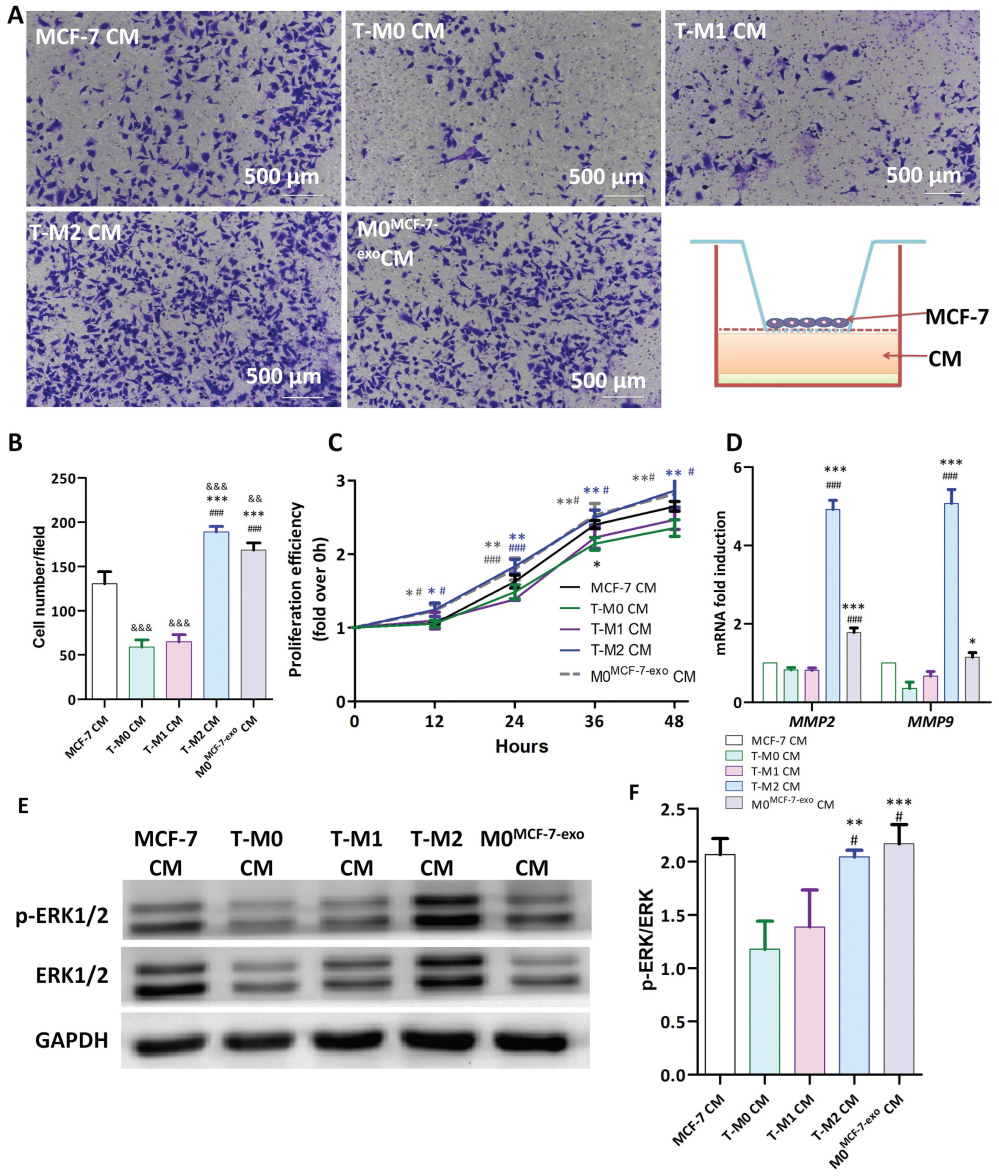
Phagocytosis of MCF-7 exosomes by human macrophages promotes the migration and proliferation of tumor cells. (**A-B**) Representative images of Transwell experiments (**A**) and counts of cells that migrated from the upper to the lower chamber (**B**). MCF-7 cells, in DMEM media without FBS, were added to the upper chamber of the Transwell apparatus, and conditioned medium from MCF-7, T-M0, T-M1, T-M2 and M0^MCF-7-exo^ cells (T-M0 cells cocultured with MCF-7 exosomes) was placed in the lower chambers for 24 hours (*** indicates statistical significance vs T-M0 CM, P<0.001; ### indicates statistical significance vs T-M1 CM, P<0.001; &&& indicates statistical significance vs MCF-7 CM, P<0.001) (n =3). (**C**) MCF-7 cells were treated with conditioned medium from MCF-7, T-M0, T-M1, T-M2 and M0^MCF-7-exo^ cells (T-M0 cells cocultured with MCF-7 exosomes) and the proliferation of MCF-7 cells was measured by the MTS assay (* indicates statistical significance vs T-M0 CM, *: P<0.05, **: P<0.005; # indicates statistical significance vs T-M1 CM, #: P<0.05, ###: P<0.001) (n =3). (**D**) Relative expression of MMP2 and MMP9 mRNAs in MCF-7 cells treated with the conditioned medium from MCF-7, T-M0, T-M1, T-M2 and M0^MCF-7-exo^ cells (* indicates statistical significance vs T-M0 CM, *: P<0.05, **: P<0.005, ***: P<0.001; # indicates statistical significance vs T-M1 CM, #: P<0.05, ##: P<0.005, ###: P<0.001) (n =3). (**E-F**) Representative images of Western blots (**E**) and the quantification of the ERK phosphorylation ratio (**F**) in MCF-7 cells treated with conditioned medium from MCF-7, T-M0, T-M1, T-M2 and M0^MCF-7-exo^ cells. p-ERK levels were normalized to total ERK levels (* indicates statistical significance vs T-M0 CM, **: P<0.005, ***: P<0.001; # indicates statistical significance vs T-M1 CM, P<0.05) (n =3).

MCF-7 exosomes promote the polarization of M0 macrophages to the M2 phenotype. Here we explored whether M0 macrophages promote tumor migration and proliferation after ingesting MCF-7 exosomes. MCF-7 cells, in DMEM media without FBS, were seeded into the upper chamber of transwell dishes and conditioned media (CM) from MCF-7 (MCF-7 CM), T-M0 (T-M0 CM), T-M1 (T-M1 CM), T-M2 (T-M2 CM) or M0^MCF-7-exo^ (M0^MCF-7-exo^ CM) was added to the lower chamber as a chemotactic substance. After 24 h of incubation, the number of MCF-7 cells that migrated was found to be significantly increased when the lower chamber of the transwell dish contained T-M2 CM and T-M0^MCF-7-exo^ CM compared to T-M0 CM. No difference in migration was detected when T-M1 CM was added. These results suggest that T-M0 cells exposed to MCF-7 exosomes have a M2-like ability and promote MCF-7 cell migration (Fig. 11A, B). To study the effects of the different CM on cell proliferation, MCF-7 cells were cultured, and the growth of these cells was measured using the CCK-8 assay. The results of this experiment showed that the proliferation of MCF-7 cells cultured in T-M2 CM and T-M0^MCF-7-exo^ CM was significantly increased compared to M0 CM, but no difference was found compared with T-M1 CM (Fig. 11C). These results show that the supernatant of T-M0 macrophages stimulated with MCF-7 exosomes could promote migration and proliferation of tumor cells and polarized T-M0 macrophages to the M2 phenotype.

To explore mechanism by which M0 macrophages stimulated by MCF-7 exosomes regulate the proliferation and migration of tumor cells, the expression and abundance of ERK signaling pathway and MMP genes and proteins were assessed in MCF-7 cells cultured in different conditioned media. Further study revealed that the mRNA levels, measured by RT-qPCR, for MMP2 and MMP9 in cells cultured in M2 CM and M0^MCF-7-exo^ CM were significantly more abundant than in cells cultured in M0 CM (Fig. 11D). As detected by Western blot, the rations of phospho-ERK 1/2 to total ERK 1/2 in cells grown in M2 CM and M0^MCF-7-exo^ CM were significantly higher than in cells cultured in M0 CM (Fig. 11E, F). These results suggest that MMP2 and MMP9 in MCF-7 cells were upregulated via activation of the ERK signaling pathway by CM from M0 macrophages stimulated by MCF-7 exosomes, thereby promoting the proliferation and migration of the tumor cells.

**Figure 12:**
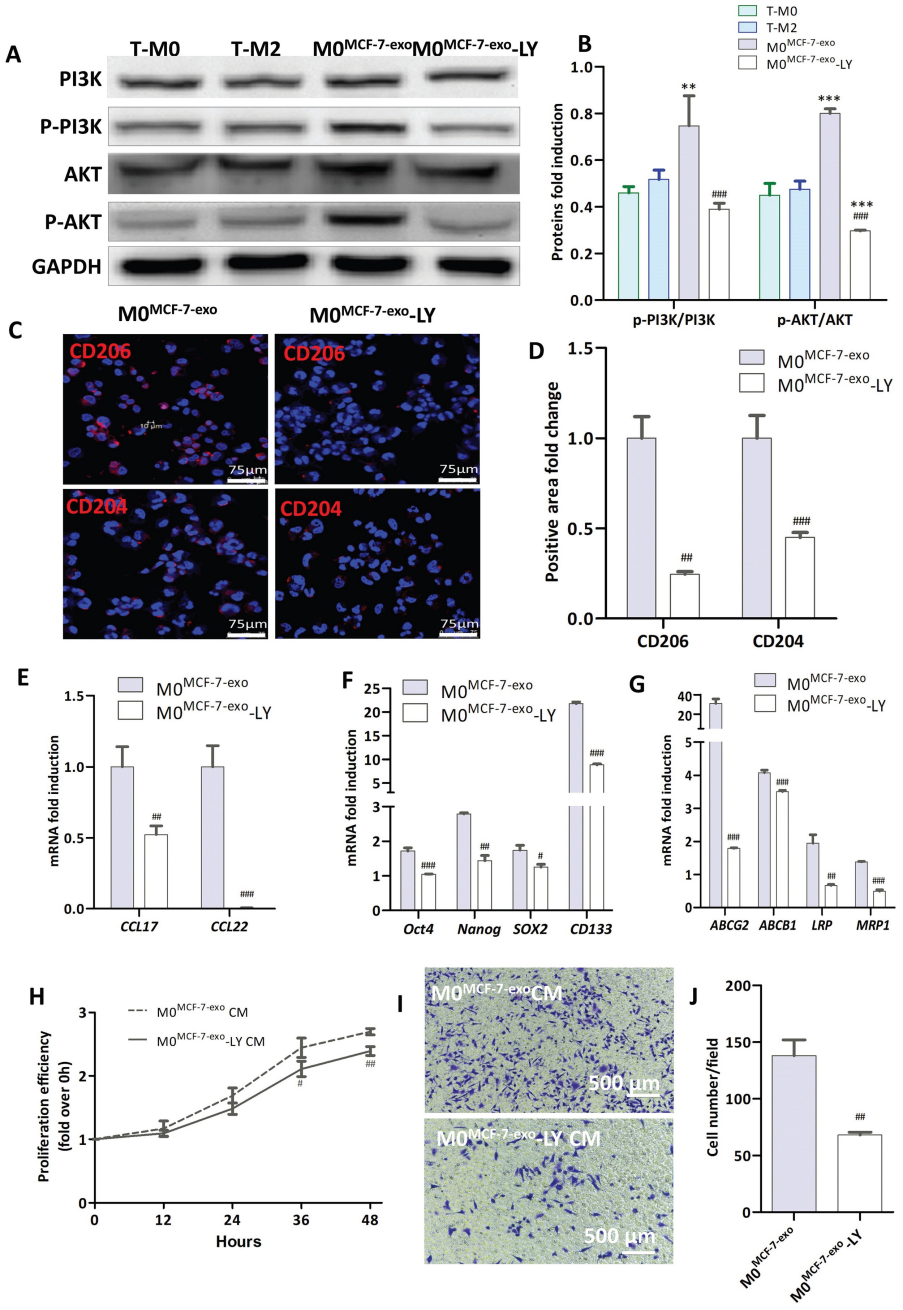
PI3K inhibitor LY294002 inhibits the migration and proliferation of tumor cells. (**A-B**) Representative Western blots (**A**) and the quantification of PI3K and Akt phosphorylation ratios (**B**). Human T-M0 and T-M1 cells were induced from THP1 cells. M0^MCF-7-exo^ cells: T-M0 cells treated with MCF-7 exosomes. M0^MCF-7-exo^-LY cells: T-M0 cells treated with MCF-7 exosomes and the PI3K inhibitor LY294002 for 48 hours. P-PI3K levels were normalized to total PI3K levels, and p-AKT levels were normalized to total AKT levels (* indicates statistical significance vs T-M0, **: P<0.005, ***: P<0.001; ### indicates statistical significance vs M0^MCF-7-exo^, P < 0.001) (n =3). (**C-D**) Representative images of immunofluorescence (**C**) and the positive area analysis (**D**) of CD206 (red) and CD204 (red) in T-M0 cells cocultured with MCF-7 exosomes with or without LY294002 (# indicates statistical significance vs M0^MCF-7-^exos, ##: P<0.005, ###: P<0.001) (n =3). (**E-G**) RT-qPCR was used to measure the abundance of mRNA for selected genes in T-M0 cells cocultured with MCF-7 exosomes with or without LY294002 (# indicates statistical significance vs M0^MCF-7-exos^, #: P<0.05, ##: P<0.005) (n =3). (**H**) Proliferation of T-M0 cells cocultured with MCF-7 exosomes with or without LY294002 measured by the MTS assay (# indicates statistical significance vs M0^MCF-7-exos^, P<0.05) (n =3). (**I-J**) Representative images of Transwell experiments (**I**) and the quantification of MCF-7 cells that migrated from the upper chamber (**J**). T-M0 cells cocultured with MCF-7 exosomes with or without LY294002 were placed in the lower Transwell chamber (## indicates statistical significance vs M0^MCF-7-exo^ CM, P<0.005) (n =3).

To explore mechanisms for the polarization of human M0 macrophages to the M2 phenotype by MCF-7 exosomes, the abundance of PI3K and Akt, and their phosphorylation status, in macrophages were determined by Western blot analysis. The results show that the ratio of phosphorylated to dephosphorylated PI3K (P<0.005) and AKT (P<0.001) in M0^MCF-7-exo^ cells were both significantly higher than in M0 macrophages (Fig. 12A, B). These results suggest that MCF-7 exosomes promote the polarization of human M0 macrophages to the M2 phenotype by activation of the PI3K/AKT signaling pathway.

To further evaluate this hypothesis, the selective PI3K inhibitor LY294002 was used to inhibit ERK signaling in M0^MCF-7-exo^ cells (M0^MCF-7-exo^-LY). As expected, the ratios of phosphorylated to dephosphorylated PI3K and AKT in M0^MCF-7-exo^-LY (P<0.001) cells were significantly lower than in M0^MCF-7-exo^ cells (Fig. 12A, B). This change is associated with significantly decreased abundances of CD206 (P<0.005) and CD204 (P<0.001), signature M2 macrophage marker proteins (Fig. 12C, D), and parallels significant decreases in the mRNA levels for the cytokines CCL17 (P<0.005) and CCL22 (P<0.001) (Fig. 12E) as observed by confocal microscopy and RT-qPCR. Interestingly, the mRNA levels, measured by RT-qPCR, for stem cell-related genes (Oct4 (P<0.001), Nanog (P<0.005), SOX-2 (P<0.001) and CD133 (P<0.001)) (Fig. 12F) and drug resistance genes (ABCG2 (P<0.001), ABCB1(P<0.001), LRP (P<0.005) and MRP1 (P<0.001)) (Fig. 12G) were significantly lower in M0^MCF-7-exo^-LY cells than in untreated M0^MCF-7-exo^ cells. Moreover, cell proliferation, measured by CCK-8, of cells cultured with M0^MCF-7-exo^-LY CM was significantly slower than for cells cultured in M0^MCF-7-exo^ CM (Fig. 12H).

The number of migrating cells identified after culturing with M0^MCF-7-exo^-LY CM (P<0.005) was also significantly reduced (Fig. 12I, J). These results support our speculation that the polarization of M0 macrophages to the M2 phenotype, and consequently their promotion of cell proliferation and migration by MCF-7 exosome stimulation, was through, at least in part, the activation of the PI3K/AKT signaling pathway.

## Discussion

As an important part of the immune system, macrophages play a key role in cancer development 25 and tissue repair 26. However, the role of macrophages in tumor metastasis is still elusive. Here we showed that some M2 macrophages (TM2, tumor-like macrophage 2) have a “change of loyalty” after they phagocytize apoptotic tumor cells, which likely plays a decisive role in tumor metastasis. Thus, distant tumor metastasis might be at least in part result from the polarization of TM2 macrophages. This hypothesis is supported by observations in this study. We showed that during tumorigenesis, monocytes were induced to differentiate from the M0 to the M2 macrophage phenotype by tumor antigens followed by their infiltration into tumor sites. The M2 macrophages would then phagocytize apoptotic tumor cells, or active components such as exosomes of tumor cells. Upon phagocytosis, changes in gene expression in the macrophages stimulated their differentiation into TM2 macrophages. Tumor related gene expression was upregulated via activation of PI3K/Akt signaling, followed by increases in cell proliferation and migration by TM2 cells, which could be effectively eliminated by knockdown of CD206. Since TM2 macrophages can circulate through the blood system they can accumulate in various parts of the body and organs, such as the liver, lung, brain and bone, due to specific receptors that attract chemotactic TM2 27. The chemotactic TM2 formed by tumor cells therefore can form metastatic tumors in distal target tissue.

Previous studies have found that monocytes, the precursor cells of M2 macrophages, are continuously present in various tissues and organs 28. Upon differentiation of monocytes into M2 macrophages, induced by tumor cells and their secreted factors or exosomes, tumor cells can then attract and reprogram M2 macrophages to support tumor growth and metastatic spread. As a part of the tumor microenvironment, M2 macrophages are chemotactically recruited by tumor cells and gather around tumor tissue 12. Studies have shown that many tumors can change the behavior of macrophages and make them support the vascularization, proliferation, invasion and metastasis of tumors 29.

When macrophages are induced to differentiate into M2 macrophages, changes in the expression of cell membrane proteins occurs 30. Images obtained by laser confocal microscopy in this study showed that CD206 was specifically overexpressed in M2 macrophages, an observation that is supported by both RT-qPCR and Western blot analysis. CD206 is an important modulator of the macrophage function, as its CTLD4-8 domain can coordinate with TLR4 to regulate the proinflammatory response induced by chitooligosaccharides 31. Our study found that upon knockdown of CD206 by siRNA in M2 macrophages differentiated from monocytes, the self-proliferative activity and the distribution of cells in cell cycle of M2 macrophages did not change significantly. However, expression and secretion of CCL17, CCL22, IL10, Arg1, Ym1, MMP2 and MMP9 decreased in parallel with the downregulation of the expression of TLR4 and MyD88 proteins and the suppression of downstream NF-κB signaling. This study also showed that the ability of M2 macrophages to promote the proliferation, metastasis, and invasion by MCF-7 cells was weakened when they were co-cultured with M2 macrophages that had been cultured in conditioned media from cells whose CD206 was downregulated by siRNA. This observation was supported by studies using *in vivo* mouse tumor models. These results indicate that CD206 modulate TAMs to promote the growth, metastasis and invasion of breast cancer cells through M2 macrophages by activation of the TLR4/MyD88/NF-κB signaling pathway.

Current cancer treatments such as radio-chemotherapy yield large numbers of apoptotic tumor cells 32. To examine the impact of phagocytosis on macrophages, macrophage cells were cocultured with MCF-7 cells to mimic a tumor microenvironment with local macrophage infiltration. Our observations demonstrate that the macrophages were activated and that they selectively phagocytize apoptotic tumor cells instead of living non-apoptotic cells. In turn, phagocytosis stimulated proliferation of macrophages and tumor progression.

Gast et al. found that macrophages can fuse with tumor cells to form fused cells that enhance the invasive and metastatic potential of tumor cells and, in turn, harm normal cells 33. Peripheral blood and ascites from ovarian cancer patients possess newly fused cells that have characteristics of both macrophage and tumor cells, as reflected by the presence of specific cell surface molecular markers for both cell types. Some studies have shown that inhibiting macrophages can reduce the occurrence of ascites in ovarian cancer patients 34. We showed in this study that the number of circulating tumor cells (CTCs) and the total amount of cfDNA, as well as their positivity rates, in ovarian cancer patients were much higher than in healthy donors. This observation indicates that CTCs and cfDNA in peripheral blood are closely associated with malignant disease. Moreover, the number of CTCs in stages I - II cancer was lower than in stages II and IV of the disease, but the total amount of cfDNA in stages I - II and IV was higher than that in stage III. These data suggest that higher levels of apoptosis and necrosis of tumor cells, or a stronger immune response, is occurring in the early stages of tumorigenesis and development. We also examined the expression of tumor-related genes in macrophages enriched from peripheral blood of ovarian cancer patients at various stages of disease. Macrophages were isolated using monoclonal antibodies linked to magnetic beads that were specific to CD86 and CD163 on the macrophages 35. These results showed that interactions between macrophages and CTCs existed at all stages of cancer. The expression of tumor-related genes in macrophages enriched from the peripheral blood of healthy volunteers was found to be almost undetectable. In contrast, the abundance of tumor markers in the cells from patients with ovarian cancer was much higher than in healthy volunteers. Interestingly, no significant difference in the abundance of the tumor-related genes HER2, MUC1, P16 and Pax8 was seen between macrophages and CTCs in the cancer patients. This data indicates that interactions between CTCs and macrophages occur in ovarian cancer patients at all stages of tumor progression, and that fused cells may be a component of the metastatic tumor cells.

Tumor cells and macrophages interact closely in the tumor microenvironment. TAMs in the tumor microenvironment and ascites induce immunosuppression and promote tumor progression and metastasis. Taking advantage of the plasticity of macrophages, by "repolarizing" M2 to M1 36, ablating TAMs in the tumor microenvironment or limiting TAM recruitment 37, can play anti-tumor roles and help reduce tumor load. By targeting CD206 or specifically delivering mRNA to M2 cells to induce M2 to M1 macrophage repolarization may promote tumor regression 38. Exosomes typically transport proteins, lipids, genetic material and other molecules from one cell to a recipient cell 39, 40. In recent years, an increasing number of studies have shown that tumor cell exosomes affect the phenotype and function of macrophages 41. We showed in our *in vitro* studies that the co-culture of M0 macrophages with MCF-7 exosomes promotes the polarization of these macrophages to the M2 phenotype. Our observations that the expression of stem cell-related and drug resistance-related genes, which are characteristic of tumor cells, in macrophages co-cultured with MCF-7 exosomes supports this hypothesis. We speculate that MCF-7 exosomes may transfer genetic material from tumors to the macrophages or activate factors in the macrophages as the exosomes are phagocytized. At the same time, M0 macrophages stimulated by MCF-7 exosomes upregulate the expression of MMP2 and MMP9 by activating ERK signaling in MCF-7 breast cancer cells, thereby promoting the proliferation and migration of tumor cells. Therefore, these “betrayed” macrophages, after phagocytosis of apoptotic tumor cell material, may serve as a helper for tumor cells and participate in tumor metastasis.

Phagocytosis of apoptotic cells by M2 macrophages is a normal process for macrophages in the clearance of damaged cells. After cancer tumor treatment, the ratio of apoptotic cancer cells to healthy cells near a tumor is typically high. M2 macrophages are not selective in clearing apoptotic cells. Monocytes, the precursor of M2 macrophages, always circulate in tissues and organs and during tumorigenesis, monocytes are induced to differentiate into M2 macrophages by the tumors. M2 macrophages infiltrate and accumulate around tumor tissue, allowing them to phagocytize apoptotic cancer cells. Active fragments of tumor DNA or RNA and exosomes will also be engulfed by these M2 macrophage cells during phagocytosis. If engulfed, information from the cancer cells could impact the behavior of M2 macrophages, leading to the disruption of the orderly regulation of gene expression, such as the activation of silenced genes. This could lead to the transformation of M2 macrophages into "TM2" macrophages. These TM2 cells can then be distributed throughout the body through the blood circulation, with these cells preferentially collecting in tissues and organs, such as lung, liver, brain and bone, which are enriched in receptors for M2 macrophages. The TM2 cells can proliferate in these locations, consequently, the “betrayed” TM2 cells can then form a distal metastatic tumor. In a previous study, our group found that CTCs could be found in the blood of patients with an early stage of ovarian cancer. At that time, we thought they were probably tumor cells from the early-stage ovarian cancer 42, but based on observations from this study, we now speculate that these “CTCs” may also have been TM2 macrophages.

TM2s may explain the clinical phenomenon of distal tumor tissue metastasis. First, because the immune system of the body cannot recognize the changes in the M2 cells, these “betrayed” TM2 can easily escape immune surveillance. The reason why cancers preferentially metastasize to lung, liver, bone and brain may be due to the presence of CCL2 and CCR2 in these tissues and organs 43, which are chemotactic for TM2 and thus, provides ideal conditions for harboring TM2. Eventually, the harbored TM2 proliferate and become, at least, part of the metastatic tumor. Second, when the number of apoptotic cancer cells is limited, as in the early stages of cancer, the probability of a change in loyalty of M2 is relatively low due to lower incidence of phagocytosis. In the middle and later stages of cancer, phagocytizing activity of macrophages on apoptotic tumor cells increases, along with increases in the number of apoptotic cancer cells, thus, the number of betrayed M2 (TM2) cells can increase. This would then be followed by an increase in the probability of distal tissue metastasis. When a patient with cancer receives chemoradiotherapy, the amount of damage to normal tissue cells should be limited by the therapeutic doses. Compared with normal cells, M2 macrophages have some characteristics of "stem cells" and are resistant to chemotherapy. While chemoradiotherapy may kill tumor cells, it cannot completely kill all of the "TM2 cells" in the body (and TM2 and M2 cells are scattered throughout the body.). Proliferation and distal metastasis to various tissues in the later stages will occur. In addition, metastatic cancers often fail to show histological features of the primary tumor. This could be because the metastatic cancer cells are due to "TM2" cells, and not primary tumor cells. Finally, it has been clinically observed that the degree of malignancy of the same types of cancer and its metastatic rate in young people are higher than that in elderly people. As the immune system of young people is often stronger than in elderly people, thus the probability of induced “betrayed” TM2 by cancer cells should be higher in younger patients than that in elderly patients, which leads to an increased probability of metastasis.

## Conclusions

**Figure 13:**
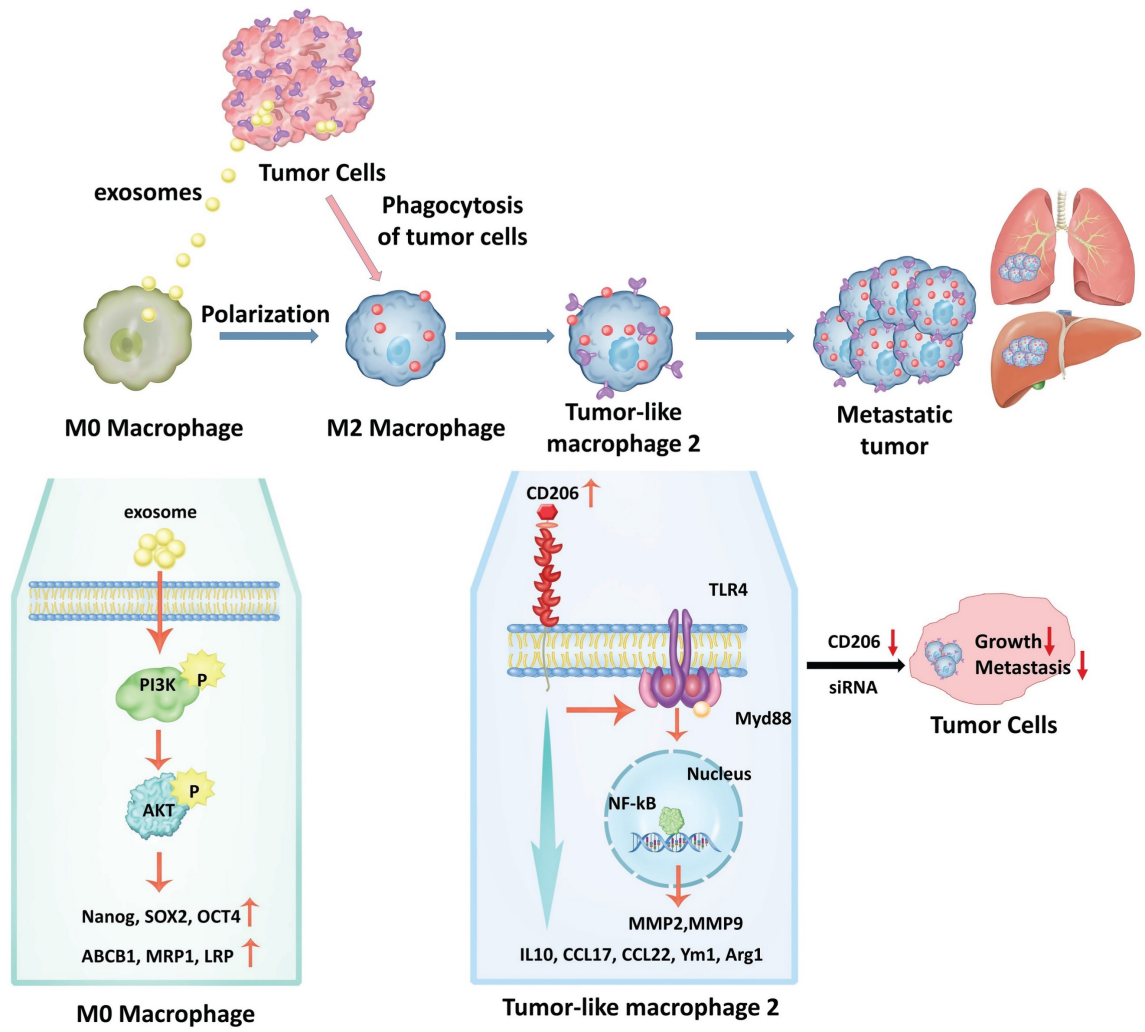
A schematic model for the molecular mechanisms associated with macrophage involvement in tumor metastasis. Phagocytosis of MCF-7 exosomes induces macrophage polarization to the M2 type via activation of PIK3/Akt signaling followed by increased expression of molecular marker genes that are characteristic of cancer stem cells, as well as genes involved in drug resistance. Upon phagocytosis of apoptotic cancer cells by M2 macrophages, these macrophages transform from M2 macrophages into TM2 cells. TM2 cells are characterized by the simultaneous expression of macrophage marker and tumor-related marker genes. Transformed TM2 cells, in turn, promote cancer cell proliferation and increase their metastatic potential, thus, together with cancer cells form a metastatic tumor. In addition, the transformation of M2 cells into TM2, and the enhanced proliferation and invasion of cancer cells, can be blocked by knockdown of CD206, which opens novel avenues for overcoming cancer metastasis.

In summary, our study showed that monocytes could be induced by tumor cells to differentiate into M2 macrophage cells. Phagocytosis of MCF-7 exosomes induced macrophage polarization to the M2 type via activation of PIK3/Akt signaling followed by increased expression of molecular marker genes that are characteristic of cancer stem cells, as well as genes involved in drug resistance. Upon phagocytosis of apoptotic cancer cells by M2 macrophages, these cells transformed into TM2 cells. TM2 cells were characterized by the simultaneous expression of macrophage marker and tumor-related marker genes. Transformed TM2 cells, in turn, promote cancer proliferation and increase metastatic potential, thus, together with cancer cells form a metastatic tumor. In addition, the transformation of M2 macrophages into TM2, and the enhanced proliferation and invasion of cancer cells, can be blocked by knockdown of CD206, which may open a novel avenue to overcome cancer metastasis (Fig. 13).

## Supplementary Material

Supplementary methods, figures and tables.
