# Supplementary material for: CD206 modulates the role of M2 macrophages in the origin of metastatic tumors
Source: J Cancer. 2024 Jan 21;15(5):1462–86. doi: 10.7150/jca.91944 (PMC10861823; doi:10.7150/jca.91944)
Supplement: Supplementary file 1 — Supplementary methods, figures and tables. [file jcav15p1462s1.pdf]

Supplementary Materials

CD206 modulates the role of M2 macrophages in the origin of metastatic tumors

Hui Li[1,†], Yingqi Miao[1,†], Liping Suo[1], Xi Wang[1], Yiqing Mao[1], Xuehui Zhang[1], Na Zhou[1], Junrui Tian[1], Xiuyan Yu[1], Tongxia Wang[2], Yan Gao[2], Hongyan Guo[2], Zheng Zhang[3], Diansheng Ma[4], Hongxia Wu[4], Yanwei Cui[4], Xiliang Zhang[4], Xiaochun Chi[5], Yuchi Li[6,*], David Irwin[7,*], Gang Niu[8,*], Huanran Tan[1,*]

†These authors contributed equally to this work and share first authorship: Hui Li, Yingqi Miao.

[*] These authors contributed equally to this work and share last authorship:Huanran Tan, Gang Niu, David Irwin.

1.Supplementary Data

1.1 Differentiation of cells into macrophages and the expression of cell surface markers

To investigate the function of M2 macrophages, THP1 human monocytes and mouse bone marrow macrophage cells were induced to differentiate into different types of macrophages using cytokines indicated in Fig. S1A & 1H. Treatment of THP1 cells with PMA, LPS or IFNγ resulted in high expression of CD68 (Fig. S1B), TNFα ($P<0.005$) and IL-1β ($P<0.001$) (Fig. S1C), which is consistent with the characteristics of M1 macrophages. Treatment of THP1 cells with PMA, IL-4 and IL-13 triggered high expression of the markers of M2 macrophages, CD206, CD204 (Fig. S1B), CCL17 ($P<0.005$) and CCL22 ($P<0.0001$) (Fig. S1D). Results of RT-qPCR ($P<0.005$ vs T-M0, $P<0.001$ vs T-M1) and Western blot ($P<0.05$ vs T-M0, $P<0.005$ vs T-M1) analyses showed that the T-M2 macrophages significantly overexpress CD206, compared to T-M0 and T-M1 macrophages (Fig. S1E, F, G).

Similarly, mouse bone marrow-derived macrophages (BMDMs) were induced to differentiate into M-M0, M-M1 and M-M2 macrophages (Fig. S1H). Flow cytometry and confocal microscopy showed that 84.5% (± 3.2%) of the Balb/c mouse bone marrow cells were viable (Fig. S1I), and the markers F4/80 and CD11b were highly expressed after induction with mMCSF for 7 days (Fig. S1J, K). These results indicate that BMDMs were successfully generated. High expression of iNOS ($P<0.001$) and IL-12 ($P<0.001$) in cells induced with LPS and IFNγ is consistent with the characteristics of M1 macrophages (Fig.S1L), and the high expression of Arg1 ($P<0.001$) and Ym1 ($P<0.005$) in IL-4-treated cells is consistent with the characteristics of M2 macrophages (Fig. S1M). Images acquired by laser confocal microscopy indicated that CD68 and CD80 were highly and specifically expressed in the M-M1 macrophages, while CD206 was specifically expressed in the M-M2 macrophages (Fig. S1N). RT-qPCR results also showed that CD206 mRNA was significantly more abundant in M-M2macrophages ($P<0.005$) (Fig. S1O). These results demonstrate that T-M2 and M-M2 cells with macrophage M2 characteristics were successfully induced, and that CD206 is a specific marker for T-M2 and M-M2 macrophages.

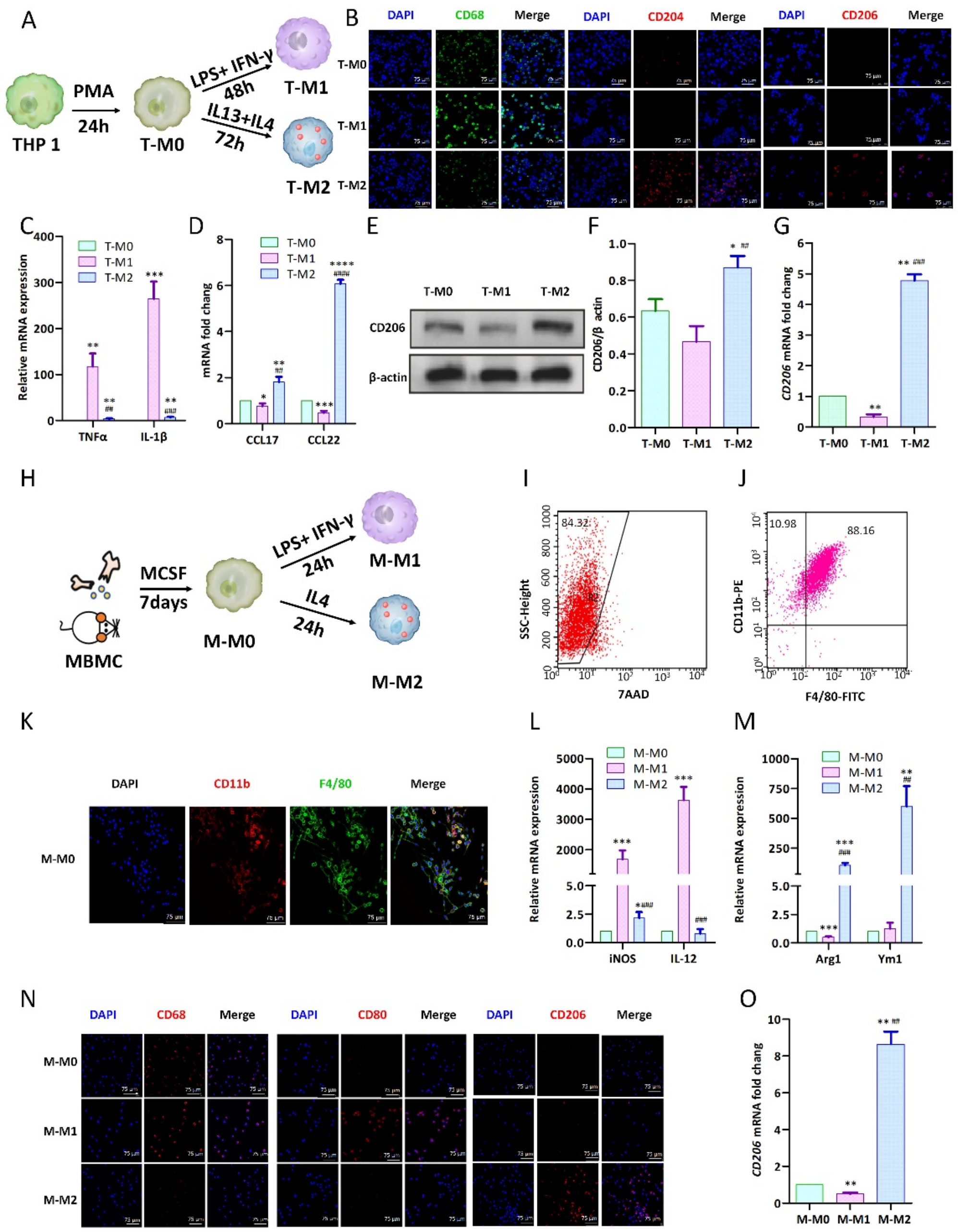

Figure S1. Differentiation of macrophages and expression of cell surface markers. (**A**)Scheme illustrating the experimental strategy for polarizing THP1 cells into macrophages. (**B**)Representative immunofluorescent images of T-M0, T-M1 and T-M2 cells after THP1 induction. CD68, CD204 and CD206 proteins were detected by immunofluorescence with primary and secondary antibodies. Nuclei were counterstained with DAPI (n = 3). (**C**)Abundance of TNFα and IL-1β mRNA were quantified by RT-qPCR in T-M0, T-M1 and T-M2 cells after induction of THP1 cells. (n = 3 independent cell cultures per group). (* indicates statistical significance vs T-M0, $P > 0.05$; # indicates statistical significance vs T-M1, $P > 0.05$) (n =3).(**D**)Abundance of CCL17 and CCL22 mRNA were quantified by RT-qPCR in T-M0, T-M1 and T-M2 cells after induction from THP1 cells. (n = 3 independent cell cultures per group). (* indicates statistical significance vs T-M0, $P > 0.05$; # indicates statistical significance vs T-M1, $P > 0.05$) (n =3).(**E-F**) Representative Western blots (e) and the quantification (f) of CD206 protein in T-M0, T-M1 and T-M2 cells induced from THP1 cells. CD206 abundance was normalized to β-actin. (n = 3 samples per group). (* indicates statistical significance vs T-M0, $P > 0.05$; # indicates statistical significance vs T-M1, $P > 0.05$) (n =3). (**G**)Abundance of CD206 mRNA in T-M0, T-M1 and T-M2 cells induced from THP1 cells. (n = 3

independent cell cultures per group). (* indicates statistical significance vs T-M0, P > 0.05; # indicates statistical significance vs T-M1, P > 0.05) (n =3).(**H**)Scheme illustrating the experimental procedure for the polarization of mouse bone marrow-derived macrophages (BMDM).(**I**)Flow cytometry of M-M0 cells 7 days after induction of harvested BMDMs with mMCSF. The R2 region represents live cells.(**J**)Flow cytometry of M-M0 cells after BMDMs were induced with mMCSF for 7 days. The upper left panel shows CD11b and F4/80 double-positive cells.(**K**)Representative immunofluorescence images of M-M0 cells after BMDMs were induced with mMCSF for 7 days.(**L**)Abundance of iNOS and IL-12 mRNA in M-M0, M-M1 and M-M2 cells induced from BMDMs quantified by RT-qPCR (n = 3) (* indicates statistical significance vs M-M0, *P* > 0.05; # indicates statistical significance vs M-M1, P > 0.05) (n =3).(**M**)Abundance of Arg1 and Ym1 mRNA in M-M0, M-M1 and M-M2 induced from BMDMs quantified by RT-qPCR (n = 3) (* indicates statistical significance vs M-M0, P > 0.05; # indicates statistical significance vs M-M1, P > 0.05) (n =3).(**N**)Representative immunofluorescence images of M-M0, M-M1 and M-M2 cells induced from BMDMs.(**O**)Abundance of CD206 mRNA in M-M0, M-M1 and M-M2 induced from BMDMs quantified by RT-qPCR (n = 3) (* indicates statistical significance vs M-M0, P > 0.05; # indicates statistical significance vs M-M1, P > 0.05) (n =3).

1.2 siRNAs transfected into M-M2 interfere with the expression of CD206

CD206 is specifically expressed by M2 macrophages, with high expression levels correlated with a poor prognosis in breast cancer. However, the role of CD206 in the promotion of breast cancer development by M2 macrophage cells remains unclear. We designed three mouse-specific siRNAs (MS1, MS2 and MS3) for transfection into mouse macrophage cells to knockdown CD206 expression. RT-qPCR results showed that the MS3 siRNA induced the greatest downregulation, compared to control (NC) siRNA, for CD206 expression in the M-M2 cells (Fig. S2A). Confocal microscopy showed that MS3 siRNA treatment for 24, 48 and 72 h led to decreased CD206 protein abundance in M-M2 macrophages (Fig. S2B), with the greatest decrease occurring at 48 hours but remaining stable through 72 hours.

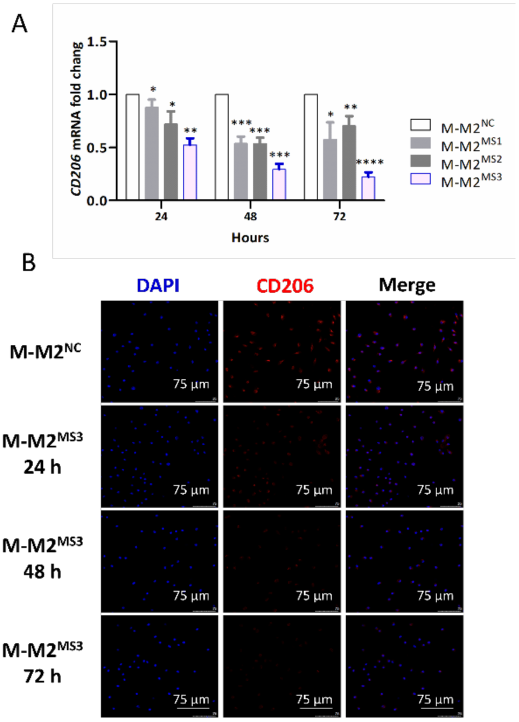

Figure S2. siRNAs transfected into M-M2 cells interfere with the expression of CD206. (**A**) CD206 siRNAs were transfected into M-M2 cells to interfere with the expression of the CD206 gene. CD206 mRNA levels were

quantified by RT-qPCR 24, 48 and 72 hours after transfection with the siRNAs (n =3). (**B**)The representative immunofluorescence confocal images of M-M2 transfected with MS3 siRNA for 48 h. Nuclei were stained with DAPI (blue) in the left column, CD206 was detected by immunofluorescence (red) in the center and the merge of the two images is shown on the right.

1.3 siRNAs transfected into T-M2 interfere with the expression of CD206

To further examine the role of CD206 in macrophages, three human-specific siRNAs (HS1, HS2 and HS3) were designed to interfere with the expression of CD206 in human T-M2 macrophages. mRNA and protein levels for CD206 in cells treated with HS1, HS2 or HS3 was found to be significantly reduced 48 and 72 hours after transfection. The knockdown activity of HS1 was strongest as seen by both RT-qPCR (Fig. S3A) and by Western blot (Fig. S3B, C) analysis, with a peak reduction observed at 48 h. The low abundance of CD206 in T-M2 macrophages was also observed by confocal microscopy at 48 h after HS1 treatment (Fig. S3D, E).

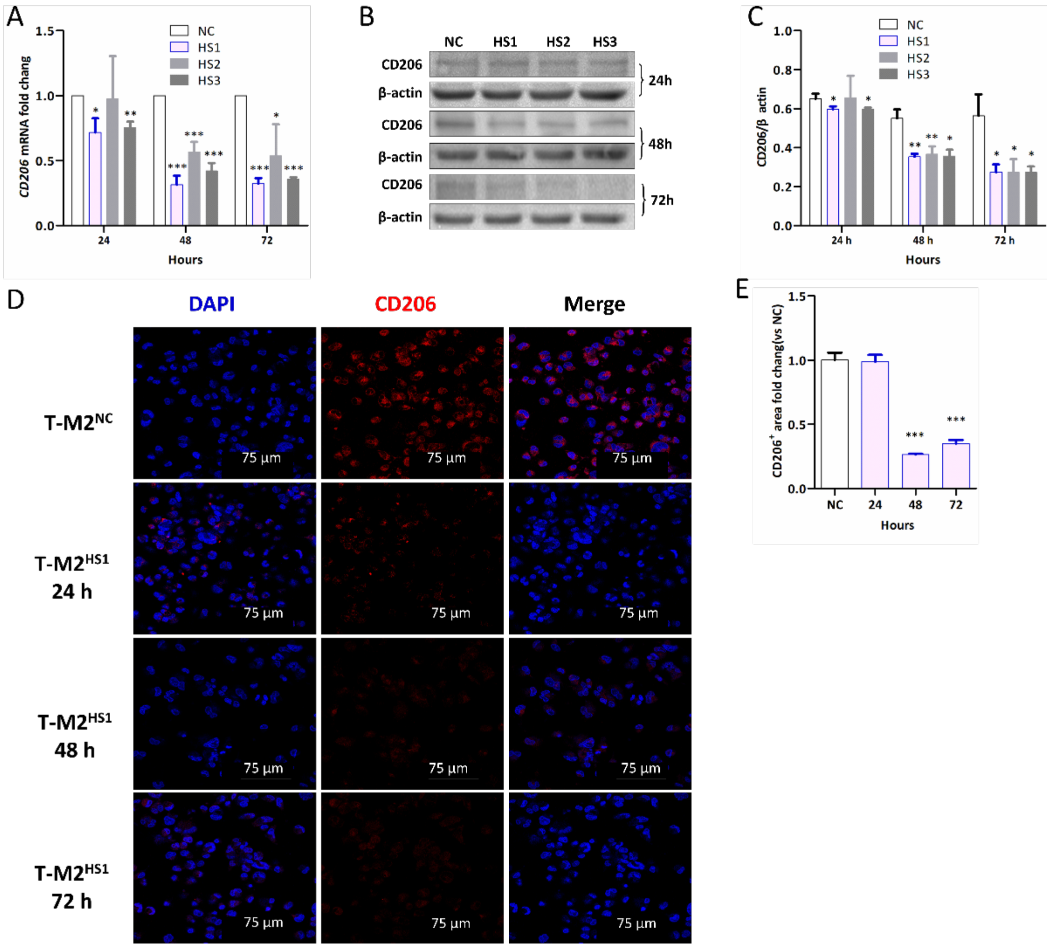

Figure S3. siRNAs transfected into T-M2 cells interfere with the expression of CD206. (**A**) siRNAs for CD206 were transfected into T-M2 cells and the abundance of CD206 mRNA was examined after 24, 48 and 72 hours. Abundance of CD206 mRNA was measured by RT-qPCR. (**B-C**) Representative western blots (**b**) and quantification (**c**) of CD206 protein levels 24, 48 and 72 hours after transfection of T-M2 cells with the NC (negative control), HS1, HS2 and HS3 siRNAs. HS1, HS2 and HS3: siRNA designed to interfere with human CD206 gene expression. (* indicates statistical significance vs NC, P<0.05) (n =3). (**D-E**) Representative immunofluorescence images (d) and analysis of fluorescence intensity (f) for CD206 (red) 48 h after transfection of T-M2 cells with HS1. (* indicates statistical significance vs NC, P<0.05) (n =3).

1.4 Characterization of MCF-7 exosomes

As an important intercellular signal transmission pathway, exosomes allow communication between tumor cells and macrophages (among other cells). To examine the effect of MCF-7 exosomes on T-M0 macrophages, an exosome uptake experiment was conducted. MCF-7 exosomes were characterized by ultracentrifugation, transmission electron microscopy and particle size analysis, which showed that they have a typical double-layer vesicle structure with a diameter of 30-200 nm (Fig. S4A, B), which is consistent with the characteristics of exosome from other sources. The exosome uptake experiment showed that DiD-labelled MCF-7 exosomes appeared in T-M0 macrophages after coculture for 48 hours (Fig. S4D). 3D reconstruction of the cells by laser confocal microscopy clearly showed that the DiD-labelled exosomes were evenly distributed in the cytoplasm of the T-M0 macrophages (Fig. S4E).

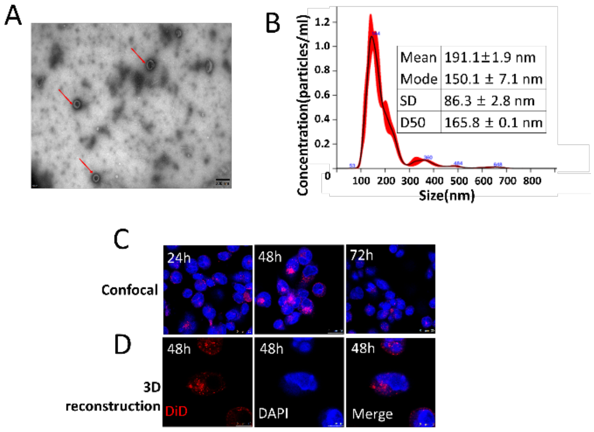

Figure S4. Characterization of exosomes from MCF-7 cells. (**A**)Transmission electron microscopy images of MCF-7 exosomes. (**B**)Particle size analysis of MCF-7 exosomes. (**C**)Representative images and 3D reconstructions generated by laser confocal microscopy of DiD-labeled MCF-7 exosomes co-cultured with T-M0 cells for 24, 48 and 72 h. (**D**)3D reconstructions generated by laser confocal microscopy of DiD-labeled MCF-7 exosomes co-cultured with T-M0 cells for 48h.

Table S1. Primers used for RT-qPCR

Gene	Species	Primer Fw	Primer Rev
4-Oct	human	GCGATCAAGCAGCGACTA	GGAAAGGGACCGAGGAGTA
ABCB1	human	GCTGGGAAGATCGCTACTGA	GGTACCTGCAAACTCTGA
ABCG2	human	TGGAATCCAGAACAGAGCTGGGGT	AGAGTTCCACGGCTGAAACACTGC
CCL17	human	AGAGCCACAGTGAGGGAGAT	TTAATCTGGGCCCTTTGTGC
CCL17	human	AGAGCCACAGTGAGGGAGAT	TTAATCTGGGCCCTTTGTGC
CCL22	human	GCGTGGTGTTGCTAACCTTCA	AAGGCCACGGTCATCAGAGT
CCL22	human	GCGTGGTGTTGCTAACCTTCA	AAGGCCACGGTCATCAGAGT
CD133	human	AGTGGCATCGTGCAAACCTG	CTCCGAATCCATTCGACGATA
CD204	human	GACGTTGGGGAGATGAGGAG	TCTGTGTCCATGAGGTTGGC
CD206	human	TGAATTGTACTGGTCTGTCCTT	GCTGTGGTGCTGTGCATTTAT
CD86	human	AGCGGCCTCGCAACTCTTAT	AAAACACGCTGGGCTTCATC
EpCAM	human	TGAGCGAGTGAGAACCTA	CACAACAATTCCAGCAAC
HER2	human	AGGAGTGCGTGGAGGAAT	AGTGGGTGCAGTTGATGG
IL-β	human	GCCAGTGAAATGATGGCTTATT	AGGAGCACTTCATCTGTTTAGG

LRP	human	TATGTGCCATCTGCCAAAGT	CATGTAGGTGCTTCCAATCA
MMP2	human	TGTGTTGTCCAGAGGCAATG	ATCACTAGGCCAGCTGGTTG
MMP9	human	CGCAGACATCGTCATCCAGT	GGACCACAACTCGTCATCGT
MRP1	human	TTGCCGTCTACGTGACCATT	AGGCGTTTGAGGGAGACACT
MUC1	human	GCACCGACTACTACCAAGAG	AAGGAAATGGCACATCACT
Nanog	human	ATGCCTCACACGGAGACTGT	AAGTGGGTTGTTTGCCTTTG
P16	human	TCTGAGAAACCTCGGGAAAC	CTCGCAAGAAATGCCCAC
PAX8	human	GAAGCAATAGCCGAGGAA	TGTAGAAAGAGCCAAGCAAA
SOX2	human	GTGAGCGCCCTGCAGTACAA	GCGAGTAGGACATGCTGTAGGTG
TNFα	human	AGCCTGTAGCCCATGTTGTA	GAGGTACAGGCCCTCTGATG
TNFα	human	AGCCTGTAGCCCATGTTGTA	GAGGTACAGGCCCTCTGATG
WT1	human	AGTCCGCCATCACAACAT	TGGTACAATAATTCCATCCC
GAPDH	human	GCACCGTCAAGGCTGAGAAC	TGGTGAAGACGCCAGTGGA
Arg	mouse	CCAACCAGGAACTGGCTGAAG	GCATCAACCCAGATGACACAGAG
Arg1	mouse	CTCCAAGCCAAAGTCCTTAGAG	GGAGCTGTCATTAGGGACATCA
CD206	mouse	AAACACAGACTGACCCTTCCC	GTTAGTGTACCGCACCCTCC
CSF2	mouse	AAGATATTCGAGCAGGGTCTACGG	CGCATAGGTGGTAACTTGTGTTTCA
Fosl1	mouse	ACCGGTCCACAGAGGTTCAT	GCCTCTCGGAGTCTGGTCTT
Hif	mouse	GGACGATGAACATCAAGTCAGCA	AGGAATGGGTTCACAAATCAGCA
IL10	mouse	GCCAGAGCCACATGCTCCTA	GATAAGGCTTGGCAACCCAAGTAA
IL12	mouse	GGAAGCACGGCAGCAGAATA	AACTTGAGGGAGAAGTAGGAATGG
IL6	mouse	CAACGATGATGCACTTGCAGA	CTCCAGGTAGCTATGGTACTCCAGA
iNOS	mouse	GCAGAGATTGGAGGCCTTGTG	GGGTTGTTGCTAACTTCCAGTC
Met	mouse	ATTGGACCCAGCAGCCTGA	TTGTCCAGCAAAGTCCCATGA
Mif	mouse	TCCGTGCCAGAGGGGTTTCTGT	ACGTTGGCAGCGTTCATGTCG
Mki67	mouse	CAGCTCCTGCCTGTTTGGAA	TCTCAGCCTCACAGGCTCATC
MMP2	mouse	CGACCACAGCCAACTACGAT	GTCAGGAGAGGCCCCATAGA
MMP9	mouse	GCTGACTACGATAAGGACGGCA	TAGTGGTGCAGGCAGAGTAGGA
NOS2	mouse	CAAGCACATTTGGGAATGGAGA	CAGAACTGAGGGTACATGCTGGAG
P105	mouse	TCCGGGAGCCTCTAGTGAGAA	TCCATTTGTGACCAACTGAACGA
Plau	mouse	GCTGTCAGAACGGAGGTGTATG	GGGCCGACCTTTGGTATCAG
Stat3	mouse	TGCACCTGATCACCTTCGAGAC	CCCAAGCATTTGGCATCTGAC
TGFβ	mouse	TACGGCAGTGGCTGAACCAA	CGGTTCATGTCATGGATGGTG
VEGF	mouse	ACATTGGCTCACTTCCAGAAACAC	TGGTTGGAACCGGCATCTTTA
Ym1	mouse	CATTCAGTCAGTTATCAGATTCC	AGTGAGTAGCAGCCTTGG
β-actin	mouse	GAAATCGTGCGTGACATTA	AGGCAGCTCGTAGCTCTT

Table S2. Complete list of antibodies used in this study

Antibody	Company	Catalogue
TLR4	Abcam, USA	ab13556
MyD88	Abcam, USA	ab133739
p65	Abcam, USA	ab32536
p-p65	Abcam, USA	ab76302

EpCAM	Abcam, USA	ab20160
CD163	Abcam, USA	ab87099
WT1	Abcam, USA	ab212951
Her2	Abcam, USA	ab134182
Pax8	Abcam, USA	ab183573
CD68	Abcam, USA	ab201973 for Human; ab283654 for Mouse
CD11b	Abcam, USA	ab197701
CD206	Abcam, USA	ab252921 for Human; ab64693 for Mouse
CD204	Abcam, USA	ab123946 for Human; ab151707 for Mouse
ERK1/2	Cell signaling technology, USA	4695
p-ERK1/2	Cell signaling technology, USA	4370
PI3K	Abcam, USA	ab191606
p-PI3K	Abcam, USA	ab182651
AKT	Abcam, USA	ab38449
p-AKT	Abcam, USA	ab81283

Table S3. Complete list of ELISA kits used in this study

ELISA kit	Company	Catalogue	Species
Arg1	Abcam, USA	ab269541	Mouse
Ym1	Boster, China	ek2010	Mouse
MMP2	Abcam, USA	ab254516	Mouse
MMP9	Sangon Biotech, China	D721181	Mouse
IL-10	Abcam, USA	ab185986	Human
CCL17	Abcam, USA	ab183366	Human
CCL22	Abcam, USA	ab223866	Human
MMP2	Sangon Biotech, China	D711194	Human
MMP9	Abcam, USA	ab246539	Human

Table S4. Abbreviations

ABCB1: ATP binding cassette subfamily B member 1, ABCG2: ATP binding cassette subfamily G member 2, AKT: Akt kinase, Arg1: arginase 1, CCL17: C-C motif chemokine ligand 17, CCL22: C-C motif chemokine ligand 22, CD204: macrophage scavenger receptor 1, CD206: mannose receptor C-type 1, CSF2: colony stimulating factor 2, EpCAM: epithelial cell adhesion molecule Fosl1: FOS like 1, Her2: erb-b2 receptor tyrosine kinase 2, Hif: hypoxia-inducible factor, IL10: interleukin 10, IL6: interleukin 6, LRP: Leucine-responsive regulatory protein, Met: MET proto-oncogene, Mif: macrophage migration inhibitory factor, MKi67: marker of proliferation Ki-67, MMP2: matrix metallopeptidase 2, MMP9: matrix metallopeptidase 9, MRP1: multidrug resistance protein, MUC1: mucin 1, MyD88: MYD88 innate immune signal transduction adaptor, Nanog: Nanog homeobox, NOS2: nitric oxide synthase 2, Oct4: organic cation/carnitine transporter4, P105: nuclear factor of kappa light polypeptide gene enhancer in B cells 1, P16: cyclin dependent kinase inhibitor 2A, P65: RELA proto-oncogene, Pax8: paired

box 8, PI3K: phosphatidylinositol 3-kinase, Plau: plasminogen activator, SOX2: SRY-box transcription factor 2, Stat3: signal transducer and activator of transcription 3, TGFβ1: transforming growth factor beta 1, TLR4: toll like receptor 4, TNFα: tumor necrosis factor alpha-like, VEGF: vascular endothelial growth factor, WT1: Wilms tumor 1, Ym1: chitinase-like 3.
